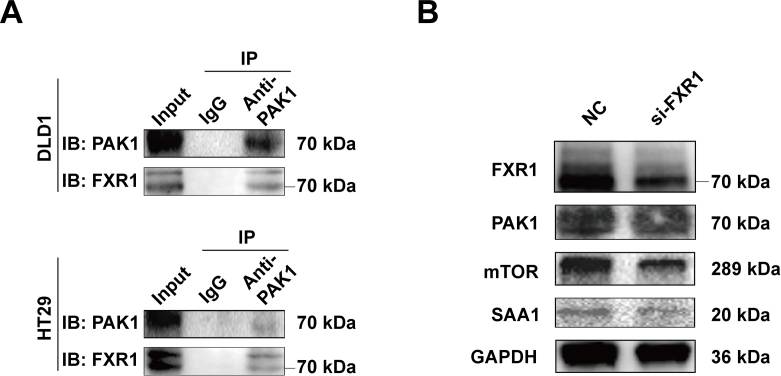# Targeting PAK1 suppresses tumor progression by promoting mRNA decay of oncogenic factors and enhancing chemotherapeutic efficacy in colorectal cancer

**DOI:** 10.1016/j.gendis.2025.101683

**Published:** 2025-05-11

**Authors:** Rongtian Pan, Renrui Zou, Ying Sui, Fei Wu, Dongfeng Wang, Yuan Zhang, Shaorong Yu

**Affiliations:** a The Affiliated Cancer Hospital of Nanjing Medical University, Jiangsu Cancer Hospital, Jiangsu Institute of Cancer Research, Nanjing, Jiangsu 210009, China; b Research Center of Clinical Oncology, Jiangsu Cancer Hospital, Jiangsu Institute of Cancer Research, The Affiliated Cancer Hospital of Nanjing Medical University, Nanjing, Jiangsu 210009, China; c Department of Medical Oncology, The Affiliated Cancer Hospital of Nanjing Medical University, Jiangsu Cancer Hospital & Jiangsu Institute of Cancer Research, Nanjing, Jiangsu 210009, China; d Department of General Surgery, Colorectal Cancer Surgery Department, The Affiliated Cancer Hospital of Nanjing Medical University, Jiangsu Cancer Hospital, Jiangsu Institute of Cancer Research, Nanjing, Jiangsu 210009, China

**Keywords:** CRC, mRNA decay, Oxaliplatin, PAK1, PF3758309

## Abstract

P21-activated kinase 1 (PAK1) plays an oncogenic role in colorectal cancer (CRC). However, the role of PAK1 in CRC progression remains incompletely understood. Here, we showed that PAK1 enhanced the mRNA stability of multiple oncogenic factors. We found that PAK1 promoted CRC initiation and progression as previously reported. Mechanistically, loss of PAK1 promoted mRNA decay and inhibited the expression of CD44, SAA1, MTOR, RPS6KB1, and EIF4G1, the factors involved in tumorigenesis in many cancers. Importantly, our results revealed that the PAK1 inhibitor, PF3758309, exhibited a profound synergistic effect with oxaliplatin in CRC. Collectively, our study unveils a novel function of PAK1 in CRC progression. Thus, these results highlight the potential of targeting PAK1 as a therapeutic strategy in CRC, particularly in combination with oxaliplatin.

## Introduction

Colorectal cancer (CRC) is a significant global health burden, comprising approximately 7% of new cancer diagnoses and accounting for 11% of cancer-related deaths.^1^ Treatment options for CRC include surgery, radiotherapy, chemotherapy, and targeted therapy. Targeted therapy aims to disrupt specific cellular signaling pathways and biological processes involved in tumor initiation and progression. The advantages of targeted therapy include high specificity, as it focuses on cancer-specific molecular targets, thus minimizing damage to normal cells and resulting in fewer side effects compared with conventional chemotherapy. Additionally, targeted therapy can be used in combination with other treatments, such as chemotherapy or radiotherapy, to enhance therapeutic efficacy.^2^ However, the limitations of targeted therapy include the development of resistance over time, as cancer cells can adapt through mutations or alternative pathways. Moreover, the high cost of targeted therapy may limit its accessibility, and its effectiveness is often restricted to patients with specific genetic mutations.^2^ These limitations underscore the need for identifying novel therapeutic targets, which may offer new avenues for overcoming the challenges associated with current therapies. Therapy targets include cell surface antigens, growth factor receptors, and key enzymes or proteins in intracellular signal transduction pathways, such as epidermal growth factor receptor (EGFR), KRAS, mitogen-activated protein kinase (MAPK), BRAF, and the phosphatidylinositol 3-kinase (PI3K)/protein kinase B (AKT)/mechanistic target of rapamycin (mTOR) pathway.^3^

The p21-activated kinase family (PAKs), as downstream target proteins of the small G protein Rho family and upstream regulatory factors of MAPK, have increasingly garnered widespread attention. PAKs regulate various signaling pathways associated with tumorigenesis, such as the PI3K/AKT pathway, c-Jun N-terminal kinase (JNK) pathway, and WNT/β-catenin pathway.^4^ PAKs belong to the serine–threonine kinase family, consisting of 6 members (PAK1–6). Initially identified as protein kinases acting downstream of Ras-related Rho-GTPases Cdc42 and Rac, PAKs are roughly divided into two groups based on their structural differences and sequence homology.^4^ These two groups consist of the group I PAKs, composed of PAK1–3, and the group II PAKs, composed of PAK4–6.

PAK1, a member of group I PAKs, plays a pivotal role in tumorigenesis. PAK1 exerts profound effects on promoting the proliferation, migration, and invasion of CRC, both *in vitro* and *in vivo*, through regulating the MAPK, PI3K/AKT, Rho GTPase, and WNT/β-catenin pathways.^5^, ^6^, ^7^, ^8^ PAK1 modulates diverse downstream signaling cascades that govern critical cellular processes such as cell adhesion, cytoskeleton reorganization, and the epithelial-to-mesenchymal transition.^9^

Furthermore, PAK1 influences the expression of key molecules that facilitate tumor invasion, notably matrix metalloproteinases, which degrade extracellular matrix components to facilitate tumor cell dissemination.^10^ PAK1 regulates CRC metastasis by requiring an extracellular-regulated kinase (ERK)-dependent phosphorylation of focal adhesion kinase (FAK) at Ser-910.^11^ Additionally, PAK1 has been shown to cooperate with hypoxia inducible factor 1 subunit alpha (HIF-1α), a transcription factor activated under hypoxic conditions within the tumor microenvironment.^12^ However, the unappreciated function and mechanism of PAK1 need to be further explored.

Messenger RNA (mRNA) decay and mRNA translation are two crucial steps of the post-transcriptional process that profoundly affect protein production.^13^ mRNA stability is widely regulated in various types of cancer.^14^ It has been reported that PAK1-dependent phosphorylation of poly(RC) binding protein 1 (PCBP1) regulates splicing and translation.^15^ In addition, active PAK1 phosphorylates fragile X-related protein 1 (FXR1), which is then recruited to stress granules.^16^ PAK1 also controlled cell shape and polarity by directly phosphorylating the RNA-binding protein Sts5 (orthologue of human DIS3L2 (DIS3-like exonuclease 2)) and then dissociated with P-bodies in fission yeast cells.^17^ These studies indicate that PAK1 might promote CRC progression through regulating mRNA decay.

This study aims to elucidate the novel oncogenic mechanism of PAK1 and to investigate the significance of PAK1 in the growth and metastasis of CRC. We specifically explored the association between PAK1 and mRNA stability. Loss of PAK1 promoted mRNA decay and inhibited the mRNA stability of multiple oncogenic factors, including cluster of differentiation 44 (CD44), serum amyloid A1 (SAA1), MTOR, ribosomal protein S6 kinase B1 (RPS6KB1), and eukaryotic translation initiation factor 4 gamma 1 (EIF4G1). Importantly, our results revealed that the PAK1 inhibitor, PF3758309 (PF-309), exhibited a profound synergistic effect with oxaliplatin (OXA) in CRC. Collectively, our study unveils a novel function of PAK1 in CRC progression and highlights the potential of targeting PAK1 as a therapeutic strategy in CRC, particularly in combination with OXA.

## Material and methods

### Chemicals and reagents

PF-309 (S7094, Selleck, China) and IPA-3 (S7093, Selleck, China) were dissolved in DMSO (BS087, Biosharp, China), aliquoted into 1.5 mL centrifugation tubes, and stored at −80 °C. OXA was procured from ApexBio Technology (A8648, ApexBio, USA). It was dissolved in dimethylformamide (D112004, aladdin, China) and stored at −80 °C.

### Cell lines and culture

We acquired six human CRC cell lines (CACO2, DLD1, HT29, SW480, HCT116, and LoVo) from the American Type Culture Collection, along with the human normal colon epithelial cell lines HCoEpiC and NCM460. HCoEpiC, NCM460, CACO2, DLD1, HT29, SW480, and HCT116 cells were cultured in Dulbecco's modified Eagle medium (KeyGEN BioTECH, China). The LoVo cell line was cultured in Ham's F12K nutrient mixture (F12K; KeyGEN BioTECH, China). All media were supplemented with 10% fetal bovine serum (WISENT CORPORATION, China), 100 U/mL penicillin, 100 μg/mL streptomycin, 250 ng/mL amphotericin B (New Cell & Molecular Biotech, China), and mycoplasma elimination reagent (YEASEN, China). Cell cultures were maintained in incubators at 37 °C with 5% CO_2_.

### Construction of stable knockout or overexpression cell lines

The PAK1 knockout plasmid was purchased from Beyotime Company (L19860, Beyotime, China). The PAK1 overexpression lentivirus and dominant negative PAK1 vector (K299R) were acquired from Corues Biotechnology in China. DLD1 and HT29 cells were subjected to PAK1 knockout, whereas HCT116 and SW480 cells were engineered for PAK1 overexpression. Stable cell lines were selected over a two-week period using puromycin at a concentration of 10 μg/mL. The efficiency of knockout and overexpression was assessed using quantitative real-time PCR (RT-qPCR) and western blotting techniques.

### siRNA design and transfection

siRNA was purchased from Ruibo Biotechnology in China. Transfection was performed using jetPRIME transfection reagent (101000046, Polyplus Transfection, France) according to the manufacturer's instructions. The most efficient siRNA sequence was selected from three candidate sequences for transfection. Table S1 presents the siRNA sequences used in this study.

### Protein and Western blotting analysis

The cell samples were treated with an immunoprecipitation lysis buffer (87787, Thermo Fisher Scientific, USA) and protease/phosphatase inhibitor cocktail (P002, New Cell & Molecular Biotech, China) at 4 °C for 30 min, followed by centrifugation at 12,000 rpm for 15 min. This protocol aims to effectively lyse the cells and extract the proteins required for further analysis. The protein concentration in each sample was determined using BCA kits (A55864, Thermo Fisher Scientific, USA), following the provided protocol to ensure uniform loading of 20 μg per sample. Subsequently, electrophoresis was conducted on a 4%–20% gradient gel (M00657, GenScript, China) in SDS-PAGE solution. Following electrophoresis, proteins were transferred onto polyvinylidene difluoride membranes (IPVH304F0, Millipore, USA) and blocked with a 5% skimmed milk powder solution. The protein-membrane complex then underwent incubation with primary and secondary antibodies, followed by visualization using enhanced chemiluminescence reagent (WBKLS0500, Millipore, USA). Table S2 shows the antibodies used in this study.

### Quantitative real-time PCR

RNA extraction was based on the EZ-10 DNAaway RNA Mini-Preps Kit (B618133, Sangon Biotech, China) and followed the manufacturer's instructions. Next, we constructed a 20 μL reverse transcription system to synthesize cDNA libraries. The system consisted of 1 μg of RNA and 4 μL of RT MIX, and was filled with DEPC water (B300592, Sangon Biotech, China) to 20 μL. The relative expression of genes was measured by quantitative PCR (Thermo Scientific, USA) using Sybr Green (Q711-02-AA, Vazyme, China). The internal parameter was GAPDH. Relative mRNA expression amounts were used by the 2^−ΔΔCt^ method. Each result was biologically replicated three times. Table S3 shows the sequence of primers used in this study.

### Cell proliferation assay

Cell viability was evaluated based on Cell Counting Kit-8 (K1018, APExBIO, USA). The number of initial cells in the 96-well plate was 4000 per well. After the addition of 10% CCK-8 to each well and following 1-h incubation, the absorbance at 450 nm was measured using a microplate reader (Bio-Rad, USA). Three duplicate wells were set up for each experiment.

### Colony formation assay

Cells were seeded into 6-well plates at a density of 300 cells per well. After three weeks of incubation to allow for colony formation, the medium was removed, and the cells were washed twice with phosphate buffer saline. Subsequently, the cells were fixed with 4% paraformaldehyde for 30 min and stained with crystal violet for 15 min. Excess stain was washed off with water, and the plates were air-dried. Colonies were then counted to determine the number of clones formed.

### Migration and invasion assay

In the migration and invasion experiment, we used transwell chambers (Corning, USA) and Matrigel (BD Biocoat, USA). In both migration and invasion experiments, fetal bovine serum-free medium containing 15,000 cells was added to the upper chamber, and 20% fetal bovine serum was added to the lower chamber. In the invasion experiment, Matrigel was diluted at a ratio of 1:10 in advance, added to the upper chamber, and together with the prepared chambers, put into the incubator until it solidified (about 30 min) for subsequent operations. After 2–3 days in the incubator, the cells were washed twice with phosphate buffer saline, then fixed with paraformaldehyde for 30 min, and dyed with crystal violet for 15 min. It was then photographed with an inverted microscope.

### SUnSET assay

Following previous experimental protocols, SUnSET assay (a non-radioactive method) was used to investigate the effect of PAK1 on mRNA translation. The cells were added to a medium containing 10 μg/mL of puromycin and placed in an incubator. After 15 or 30 min, the cells were collected and the proteins were extracted for western blotting. Relative changes in protein synthesis were measured by the reactivity of different cells against anti-puromycin monoclonal antibody (MABE343, Millipore, USA).

### Actinomycin D assay

Cells were seeded at a density of 200,000 cells per well in a 6-well plate. After cell adhesion, Actinomycin D (HY-17559, MCE, China) was added to a final concentration of 1 μg/mL. Cells were harvested at 1, 3, and 6 h post-treatment, and RNA was extracted for reverse transcription and RT-qPCR.

### DIA proteomics analysis

The liquid chromatography-tandem mass spectrometry analysis was conducted using a U3000 UHPLC system and an Orbitrap Exploris 480 (Thermo Scientific). Peptides dissolved in 0.1% formic acid were initially loaded onto a trap column. The eluent was then transferred to a reversed-phase analytical column (75 μm × 500 mm, 2 μm). The elution gradient was set from 1% to 35% buffer B (0.1% formic acid in 99.9% acetonitrile) at a flow rate of 1.2 μL/min over 90 min. The mass spectrometry parameters were configured with a full scan resolution of 120,000 within a 350–1200 *m*/*z* range, an automatic gain control target of 3e6, and an injection time of less than 50 ms. DIA data files were processed using Spectronaut (17.2.230208.Quasar) with the directDIA method. Differentially expressed proteins were identified, characterized by an absolute log_2_ fold change greater than 1 and a *p*-value less than 0.05 based on the proteomics analysis.

### Computational docking of PF-309 with PAK1

The crystal structure of PAK1 protein used for the docking was downloaded from the PDB database (PDB ID 3FXZ), the 3D structure of the small molecule PF-309 was downloaded from the PUBCHEM database, and the energy was minimized under the MMFF94 force field. In this study, AutoDock Vina 1.1.2 software was used to perform molecular docking. Before docking, PyMol 2.5.5 was used to treat receptor proteins, including removing water molecules, salt ions, and small molecules. The docking box was then set up to enclose the entire protein structure. In addition, ADFRsuite 1.03 was used to convert all processed small molecules and receptor proteins into the PDBQT format required for AutoDock Vina 1.1.2 docking. For interconnection, the global search detail was set to 32, and the default settings were retained for other parameters. The docking conformation with the lowest output score was considered to be the associative conformation. Finally, PyMol 2.5.5 docking results were used for visual analysis.

### Synergy determination with SynergyFinder

Cells were seeded into a 96-well plate at a density of 4000 cells per well. Subsequently, a combination of PF-309 and OXA was applied after dilution to gradient concentrations based on the IC_50_ values of the cells to these drugs. After 3 days, cell viability was measured using the CCK8 assay (as described above). The SynergyFinder software (https://synergy-finder.fimm.fi) was then used to calculate the drug combination synergy score. The highest single agent (HSA) method was employed, where an HSA score greater than 0 indicated synergy, and an HSA score greater than 5 indicated strong synergy.

### Co-immunoprecipitation assay

Cells were collected, washed, and lysed with an immunoprecipitation lysis buffer (87787, Thermo Fisher Scientific, USA) containing protease and phosphatase inhibitors. A small portion of the lysate was reserved as input. Protein A/G magnetic beads (88802, Thermo Fisher Scientific, USA) were washed and incubated with 5 μg of specific antibody (or IgG control) at 4 °C for 4 h. After incubation, the beads were washed and incubated overnight with the cell lysates. The next day, the beads were washed, resuspended in 2 × loading buffer, and heated at 100 °C for 10 min, and the supernatant was analyzed by SDS-PAGE alongside the input samples.

### Gene set enrichment analysis (GSEA)

We performed GSEA using a merged dataset from the TCGA COAD and READ cohorts, with PAK1 expression as the reference phenotype. The analysis was conducted with the Hallmark gene set from MSigDB (version 2023.2). We used the GSEA software with 1000 permutations, a weighted scoring scheme, and Pearson correlation as the ranking metric. The gene sets included had a minimum size of 15 and a maximum size of 500. From the top 20 enriched gene sets, we selected two significant ones for visualization.

### Organoid culture and drug sensitivity assays

Human CRC organoids were cultured following standard protocols. Briefly, CRC tissues were processed and digested using a tissue digestion solution (K601008, bioGenous, China) to isolate organoids. The resulting cells were embedded in Matrigel and cultured in an organoid basal medium (B213152, bioGenous, China) under standard conditions (37 °C, 5% CO_2_). Organoids were passaged every 7–14 days.

For drug combination assays, organoids were treated with the indicated drugs, and the effects of the drug combinations were observed. After 2 days of treatment, images were captured using a Zeiss microscope to document morphological changes. Cell viability was assessed using the enhanced ATP assay kit (S0027, Beyotime, China), following the manufacturer's instructions.

### Xenograft model and drug treatment

Five-week-old female BALB/c nude mice (13–14 g) were purchased from Vital River Laboratories and maintained under specific pathogen-free conditions. HCT116 human CRC cells (5 × 10^6^) were suspended in a mixture of phosphate buffer saline and 25% Matrigel (0827245, ABW, China) and subcutaneously injected into the right flanks of the mice. 23 days later, mice were randomly assigned to four groups (*n* = 5 per group) and received intraperitoneal injections of phosphate buffer saline, PF-309 (7.5 mg/kg), OXA (3 mg/kg), or a combination of PF-309 and OXA, administered every other day for five doses. Tumor volumes and body weights were measured every other day. On day 33, tumors were harvested, weighed, and analyzed. Tumor tissues were collected and processed for RNA and protein extraction to allow further analyses.

### Statistical analysis

Statistical analyses were performed using Prism 10.0 (GraphPad, La Jolla, USA) and the online SynergyFinder software. The data were presented as mean ± standard deviation, and all experiments and analyses were conducted in triplicate. Statistical significance was determined using student's *t*-test, unpaired *t*-test, and one-way ANOVA, considering the test of homogeneity of variances. *p*-values less than 0.05 were considered statistically significant (ns, no significance; ∗*p* < 0.05, ∗∗*p* < 0.01, and ∗∗∗*p* < 0.001).

## Results

### PAK1 deficiency inhibits CRC progression

To investigate the role of PAK1 in CRC, we assessed its expression levels across various CRC cell lines using RT-qPCR and western blotting. As shown in Figure 1A and B, CRC cell lines exhibited significantly higher PAK1 expression than normal intestinal epithelial cells HCoEpiC and NCM460. Immunohistochemical analysis from the Human Protein Atlas (HPA) database further confirmed elevated PAK1 expression in CRC tissues (Fig. 1C). To explore PAK1's functional role, we generated CRISPR/Cas9-mediated PAK1 knockout (KO) and lentiviral-mediated PAK1 overexpression (OE) cell lines. RT-qPCR and Western blotting confirmed the efficiency of PAK1 KO and OE. In DLD1 and HT29 cells, PAK1 KO significantly reduced PAK1 mRNA and protein levels compared with PAK1 wild-type (WT) (Fig. S1A, B). In HCT116 and SW480 cells, PAK1 OE significantly increased PAK1 mRNA and protein compared with the negative control (NC) (Fig. S1C, D). Furthermore, in HT29 and DLD1 cells, PAK1 KO significantly reduced *in vitro* proliferation, colony formation, and motility (migration/invasion) compared with WT cells (Fig. 1D–F). Conversely, in HCT116 and SW480 cells, PAK1 OE led to a significant increase in proliferation, colony formation, and motility compared with NC (Fig. S1E–G). These results demonstrate that targeting PAK1 inhibits CRC progression by reducing proliferation, migration, and invasion *in vitro*.

**Figure 1:**
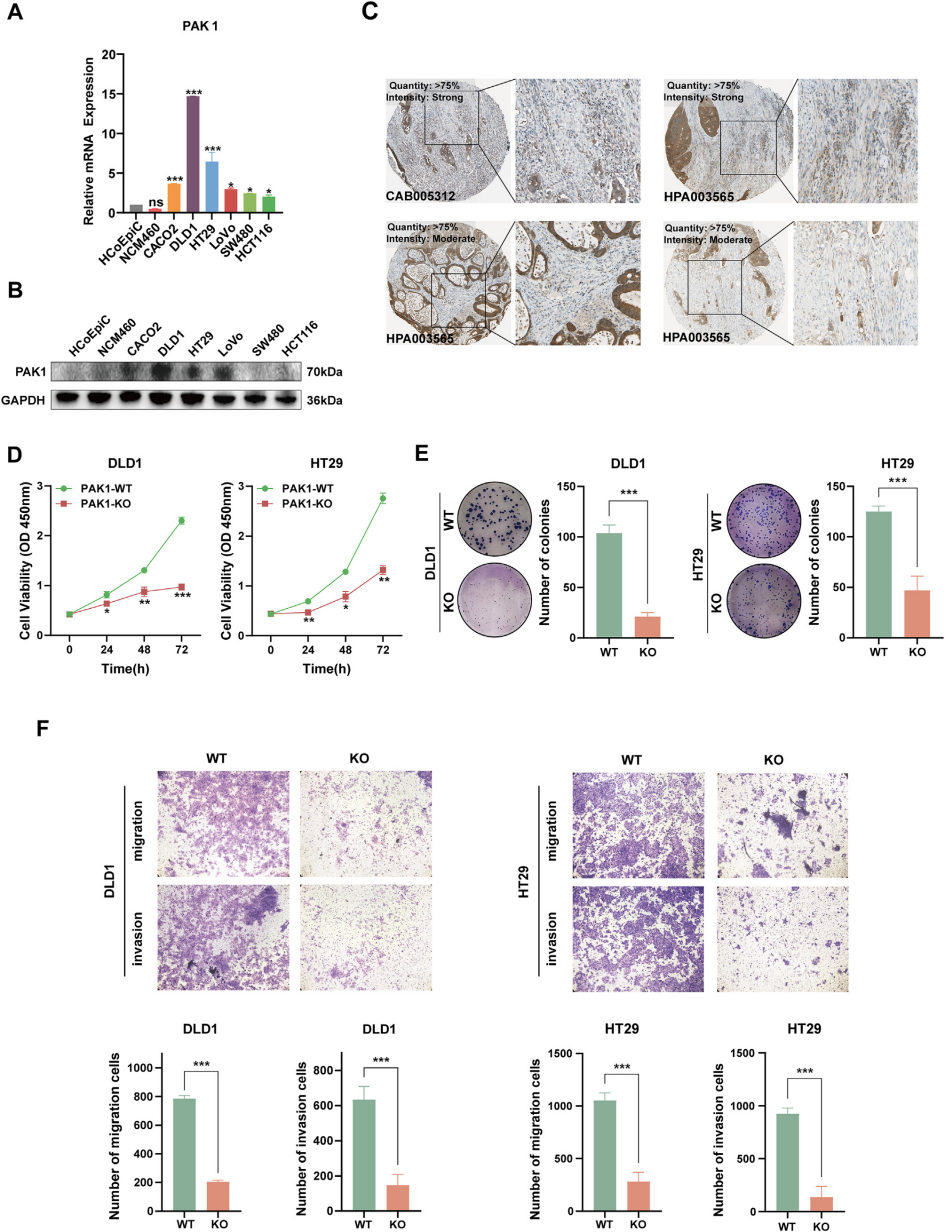
PAK1 deficiency inhibits CRC progression. **(A)** Relative mRNA expression levels of PAK1 in CRC cell lines (CACO2, DLD1, HT29, LoVo, SW480, and HCT116) compared with normal intestinal epithelial cells (HCoEpiC and NCM460). **(B)** Western blotting analysis of PAK1 protein levels in CRC cell lines and normal intestinal epithelial cells (HCoEpiC and NCM460). **(C)** Immunohistochemical staining of PAK1 in CRC tissues from the Human Protein Atlas (HPA) database. **(D)** Proliferation assays of DLD1 and HT29 cells with PAK1 knockout (KO) compared with wild-type (WT) cells. Cell viability was measured using the CCK8 assay at different time points (0, 24, 48, and 72 h). **(E)** Colony formation assays of DLD1 and HT29 cells with PAK1 KO compared with WT cells. **(F)** Migration and invasion assays of DLD1 and HT29 cells with PAK1 KO compared with WT cells. The data were presented as mean ± standard deviation, with statistical significance indicated (ns, no significance; ∗*p* < 0.05, ∗∗*p* < 0.01, and ∗∗∗*p* < 0.001).

### PAK1 activates the mTOR-S6K pathway in CRC

To investigate the role of PAK1 in CRC, we conducted GSEA. Figure 2A presents the GSEA results, which revealed significant enrichment in pathways related to PI3K/AKT/mTOR signaling and mTORC1 signaling in samples with high PAK1 expression. These findings suggest that PAK1 plays a pivotal role in these signaling pathways, which are crucial for cancer progression. Considering the crucial role of mTOR in the regulation of translation, we conducted the SUnSET assay. Figure 2B shows the translation rates in WT and KO DLD1 (left) and HT29 (right) cell lines after 15 min and 30 min of puromycin treatment. Puromycin incorporation was detected via immunoblotting, demonstrating that PAK1 KO significantly reduces the translation rate in DLD1 and HT29 cells.

**Figure 2:**
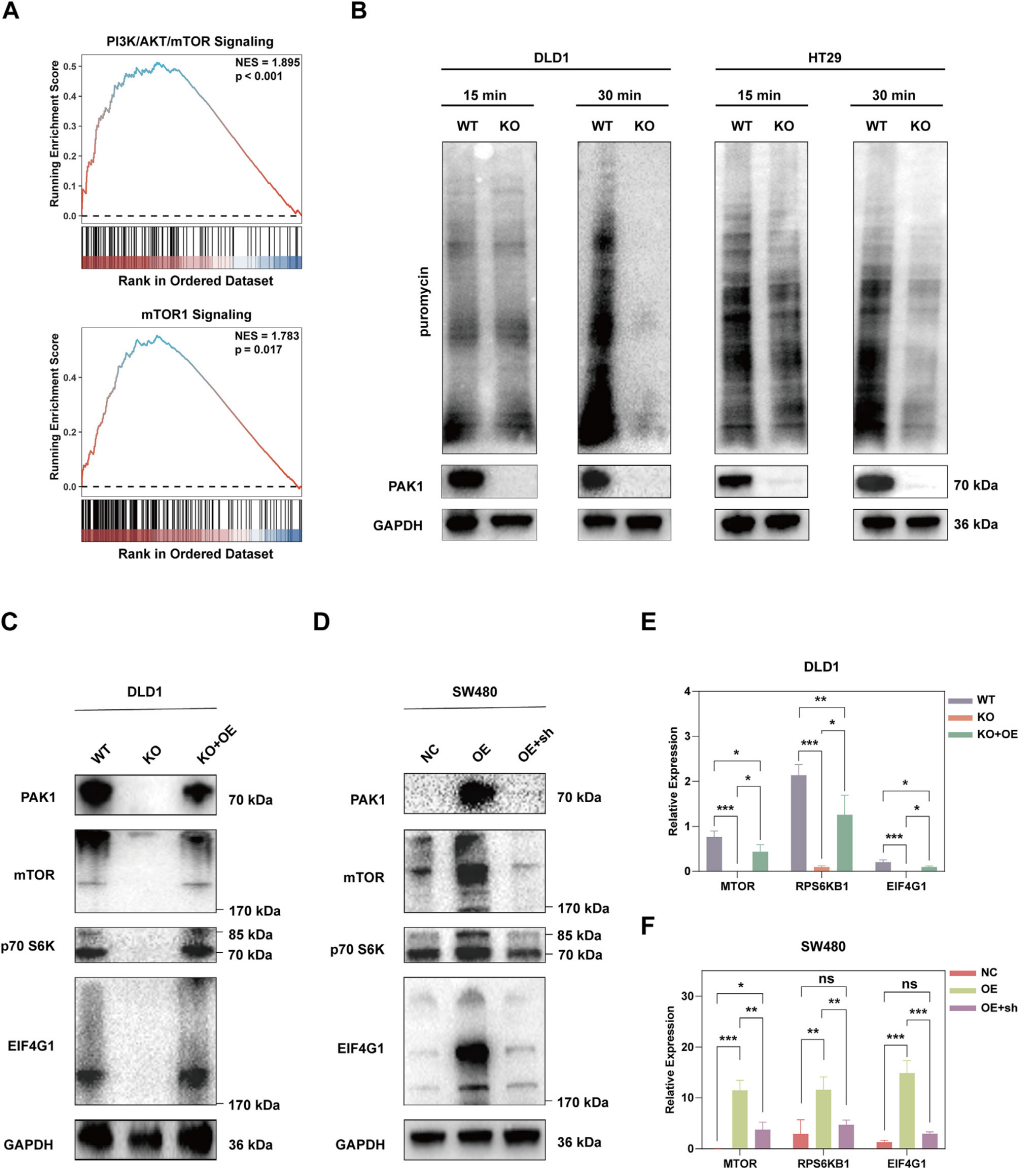
PAK1 activates the mTOR-S6K pathway in CRC**. (A)** GSEA results showed significant enrichment in pathways related to PI3K/AKT/mTOR signaling and mTORC1 signaling in PAK1 high-expression samples. **(B)** SUnSET assay showed the translation rates in wild-type (WT) and PAK1 knockout (KO) DLD1 (left) and HT29 (right) cell lines after 15 min and 30 min of puromycin treatment. Puromycin incorporation was detected via immunoblotting. **(C)** Western blotting analysis showed that PAK1 KO in DLD1 cells resulted in a significant decrease in the expression of mTOR, p70 S6K, and EIF4G1 proteins. Re-expressing PAK1 in KO cells restored the expression of mTOR, p70 S6K, and EIF4G1 to levels similar to those observed in WT cells. **(D)** Western blotting analysis showed that PAK1 OE in SW480 cells led to increased expression of mTOR, p70 S6K, and EIF4G1 proteins. Knocking down PAK1 in OE cells using shRNA reduced the expression of these proteins to levels similar to those observed in NC cells. **(E, F)** Quantitative real-time PCR results showed the relative expression of MTOR, RPS6KB1, and EIF4G1 in DLD1 and SW480 cells. The data were presented as mean ± standard deviation, with statistical significance indicated (ns, no significance; ∗*p* < 0.05, ∗∗*p* < 0.01, and ∗∗∗*p* < 0.001).

To validate the molecular mechanisms underlying PAK1's role in CRC, we examined the expression of key proteins in the mTOR signaling pathway. PAK1 deficiency in DLD1 cells resulted in a significant decrease in the protein expression of mTOR, p70 S6K, and EIF4G1, while the rescue assay by the reintroduction of PAK1 in PAK1 KO DLD1 cells led to increased expression of these proteins (Fig. 2C). Additionally, knocking down PAK1 in OE cells using shRNA also reduced the expression of these proteins to levels similar to those observed in NC cells (Fig. 2D). Unexpectedly, we found that PAK1 also regulated mRNA expression of MTOR, RPS6KB1, and EIF4G1 (Fig. 2E, F). Our findings indicate that PAK1 may promote CRC progression through the mTOR-S6K pathway. Furthermore, the puromycin signal in OE-PAK1 cells remained consistent even with mTOR inhibition (Fig. S2), suggesting that PAK1's effect on global protein stability is not entirely dependent on mTOR pathway activity.

### PAK1 promotes the mRNA stability of multiple oncogenic factors in CRC cells

To further elucidate the mechanism of PAK1 in CRC progression, we performed a data-independent acquisition (DIA) proteomic analysis, which aimed to investigate differentially expressed proteins in WT and PAK1 KO DLD1 cells. The volcano plot in Figure 3A illustrates the most significantly down-regulated proteins in PAK1 KO cells, with PRSS3, CD44, PROS1, and SAA1 showing the greatest decrease (Fig. 3B). Furthermore, we confirmed that PAK1 KO resulted in a marked decrease in CD44 and SAA1 protein levels (Fig. 3C, D). Consistent with the above findings, PAK1 KO significantly inhibited mRNA expression of CD44 and SAA1 (Fig. 3E, F). PAK1 was reported as a regulator of P-body and stress granule,^17^ thus, we assumed that PAK1 promoted CRC progression through mRNA decay.

**Figure 3:**
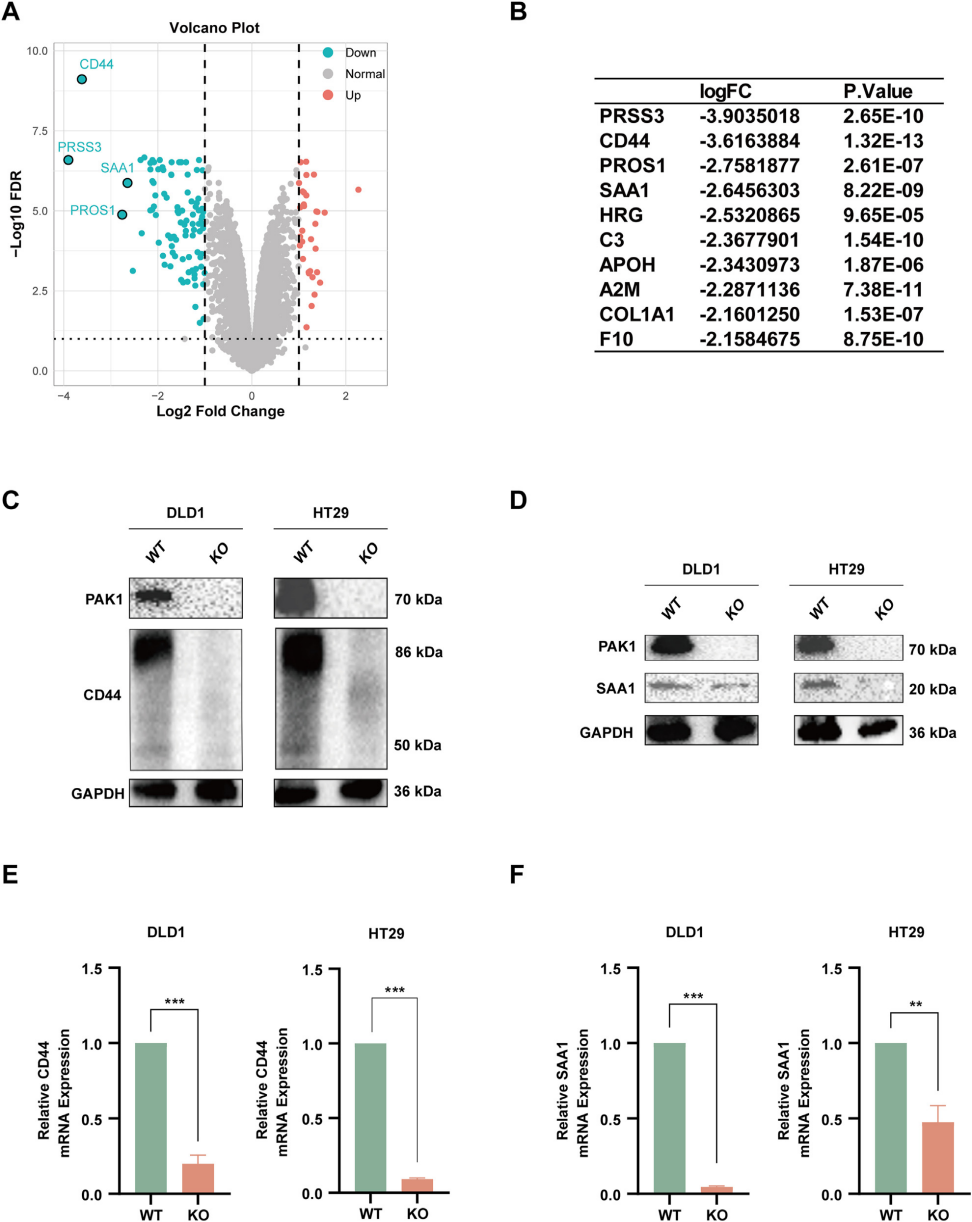
PAK1 promotes mRNA stability of multiple oncogenic factors in CRC cells. **(A)** The volcano plot shows the significantly down-regulated proteins in PAK1 knockout (KO) cells compared with wild-type (WT) cells. **(B)** The table lists the most significantly down-regulated proteins, with PRSS3, CD44, PROS1, and SAA1 being the most notable. **(C)** Western blotting validation of CD44 protein levels in WT and PAK1 KO cells. A significant decrease in CD44 protein is observed in PAK1 KO cells. **(D)** Western blotting analysis demonstrated decreased SAA1 protein levels in PAK1 KO cells. **(E)** Quantitative real-time PCR results confirmed reduced CD44 mRNA expression in PAK1 KO cells compared with WT cells. **(F)** Quantitative real-time PCR results showed reduced SAA1 mRNA levels in PAK1 KO cells compared with WT cells. The data were presented as mean ± standard deviation, with statistical significance indicated (∗∗*p* < 0.01 and ∗∗∗*p* < 0.001).

To demonstrate our hypothesis, we conducted actinomycin D experiments to assess mRNA stability. The results (Fig. 4A–C) showed that PAK1 KO cells had enhanced mRNA degradation rates for MTOR, CD44, and SAA1 compared with WT cells, suggesting a role for PAK1 in mRNA stabilization. However, no such effect was observed for U6, PAK2, PAK4, PAK5, and PAK6 (Fig. 4D–H). Thus, our results suggested the specific function of PAK1 in the mRNA stability of oncogenic genes.

**Figure 4:**
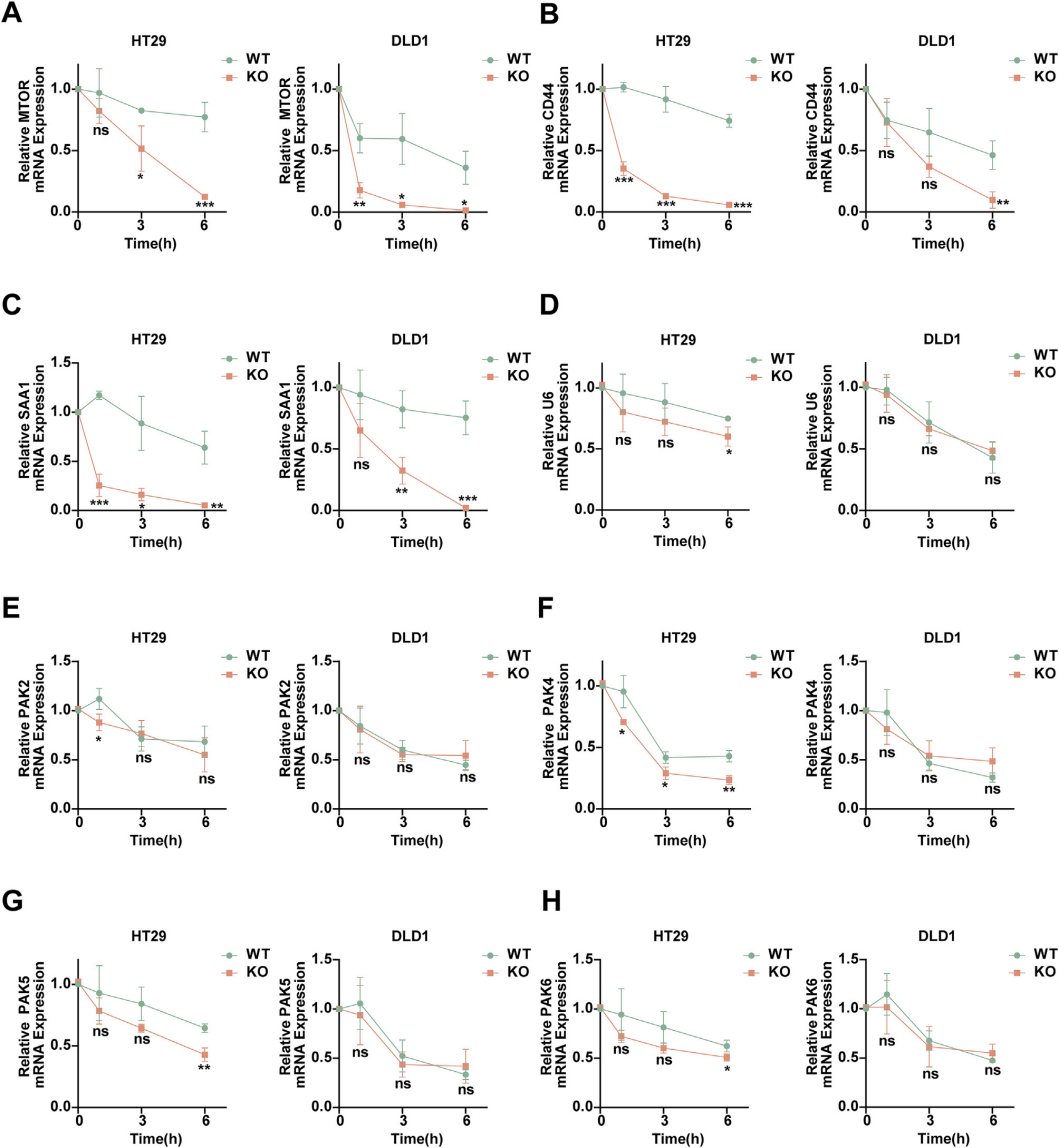
PAK1 inhibits mRNA decay of multiple oncogenic factors in CRC cells. **(A**–**H)** Relative mRNA expression levels of MTOR (A), CD44 (B), SAA1 (C), U6 (D), PAK2 (E), PAK4 (F), PAK5 (G), and PAK6 (H) in wild-type (WT) and knockout (KO) HT29 and DLD1 cells at 1, 3, and 6 h after actinomycin D treatment. The data were presented as mean ± standard deviation, with statistical significance indicated (ns, no significance; ∗*p* < 0.05, ∗∗*p* < 0.01, and ∗∗∗*p* < 0.001).

### Targeting PAK1 with PF-309 inhibits key signaling pathways in CRC cells

To assess the efficacy of PF-309, we first determined its half-maximal inhibitory concentration (IC_50_) in two normal intestinal epithelial cell lines and six CRC cell lines. As shown in Figure S3A, PF-309 exhibited varying IC_50_ values across the cell lines, indicating differential sensitivity to the inhibitor. Next, we investigated the impact of PAK1 KO on the sensitivity to PF-309 in DLD1 and HT29 cells. Figure 5A and B demonstrate that PAK1 KO led to a significant increase in IC_50_ compared with WT cells, suggesting that PAK1 plays a crucial role in mediating the response to PF-309 in these cell lines.

**Figure 5:**
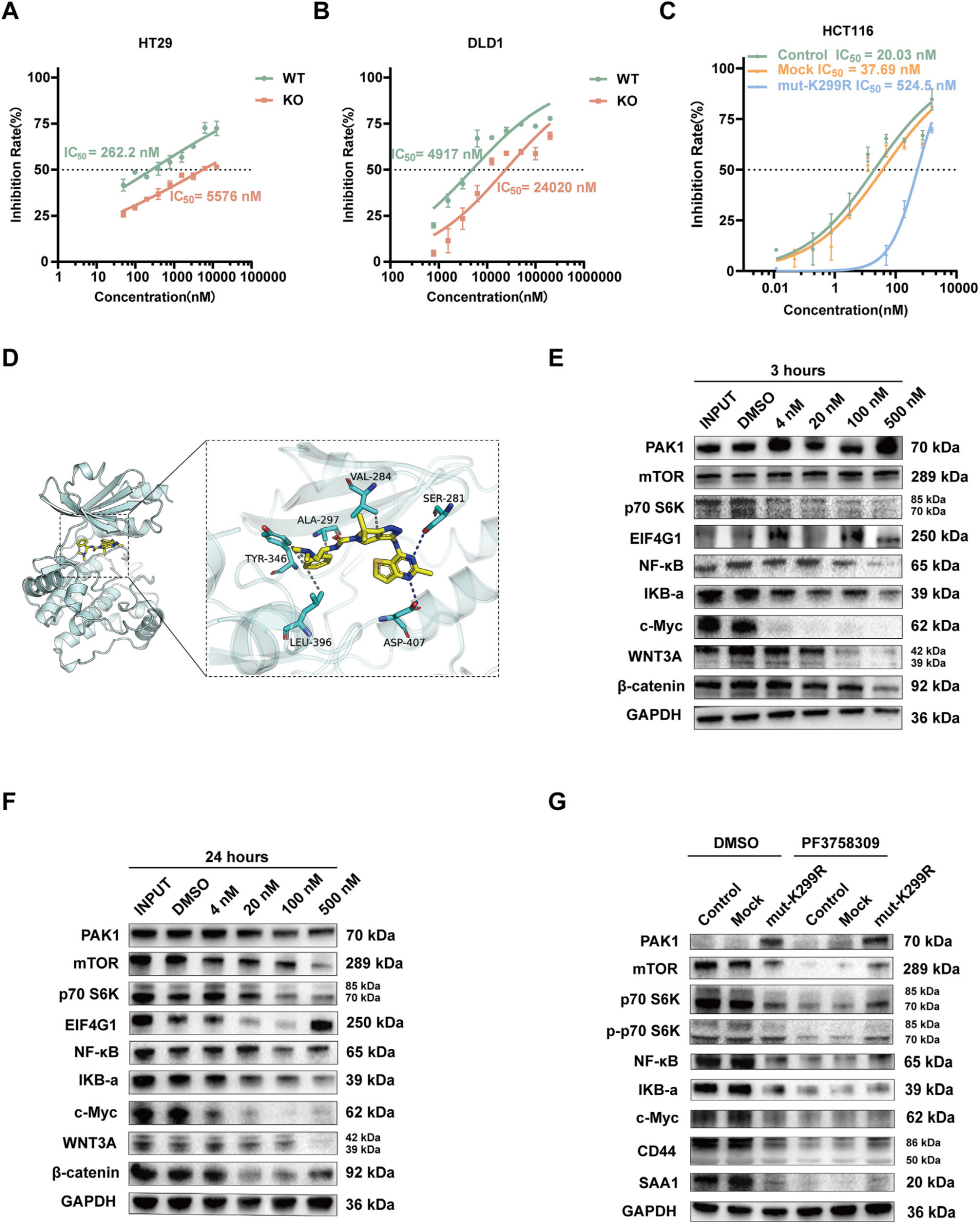
Efficacy of PF3758309 (PF-309) in CRC cell lines and its impact on signaling pathways. **(A, B)** Impact of PAK1 knockout (KO) on the sensitivity to PF-309 in DLD1 (A) and HT29 (B) cells. PAK1 KO led to a significant increase in IC_50_ compared with wild-type (WT) cells. **(C)** Dose–response curves for PF-309 in HCT116 cells expressing Control, Mock, and mut-K299R construct. The mut-K299R construct showed a significantly higher IC_50_ compared with other groups, indicating reduced sensitivity to PF-309 when PAK1 kinase activity is disrupted. **(D)** Based on the binding mode of PF-309 and PAK1 obtained by docking, the left picture is the overall view, and the right picture is the local view. In the figure, the yellow stick is a small molecule, the cyan cartoon is a protein, the blue line represents hydrogen bonding, and the gray dashed line represents hydrophobic action. **(E, F)** Western blotting analysis of HCT116 cells treated with PF-309 explored its impact on various signaling pathways. Treatment for 3 h (E) and 24 h (F) with PF-309 resulted in the inhibition of several key pathways, including mTOR, p70 S6K, EIF4G1, NF-κB, IKB-α, c-Myc, WNT3A, and β-catenin. **(G)** Western blotting analysis of HCT116 cells treated with DMSO or PF-309, comparing Control, Mock, and mut-K299R groups. The mut-K299R group displayed minimal changes in key signaling pathways, including mTOR, p70 S6K, p-p70 S6K, NF-κB, IKB-α, c-Myc, CD44, and SAA1, following PF-309 treatment, highlighting the importance of intact PAK1 function for PF-309's efficacy.

The interaction between the small molecule PF-309 and the PAK1 protein is shown in Figure 5D. It can be seen from the figure that PF-309 forms hydrogen bonds with SER-281 and ASP-407 on PAK1 protein, and salt bridges with GLU-190. The formation of hydrogen bonds and salt bridges allows proteins to bind more tightly to small molecules. p-pi interaction with LYS-274 on the protein, and hydrophobic interaction with ALA-297, VAL-284, TYR-346, and LEU-396 provide the molecule with a strong van der Waals force.

To validate the specificity of ATP-competitive inhibitor PF-309 and investigate its mechanism, we constructed a dominant-negative PAK1 (kinase-dead mut-K299R) vector. K299 of PAK1 is essential for ATP binding and kinase activity, making it a critical site for PAK1's function.^18^ As is shown in Figure 5C, the IC_50_ value for PF-309 treatment in HCT116 cells significantly increased by more than 26-fold in the mut-K299R group, compared with the control and mock groups, indicating that the K299R mutation reduces the cells' sensitivity to PF-309. These results suggest that the kinase domain of PAK1 compromised the drug effectiveness of PF-309.

Treatment with PF-309 for 3 h (Fig. 5E), 6 h (Fig. S3B), 12 h (Fig. S3C), and 24 h (Fig. 5F) in WT cells consistently inhibited multiple pathways, including mTOR-S6K, NF-κB, c-Myc, and β-catenin, demonstrating its broad-spectrum anti-tumor effects. In contrast, in the mut-K299R group (Fig. 5G), these key signaling pathways were slightly affected even after 24 h of PF-309 treatment, further confirming the importance of PAK1 integrity for PF-309's action. These evidences support PF-309's potential as an effective CRC therapy through targeting PAK1, as its anti-tumor effects are significantly diminished when PAK1 function is compromised.

### PF-309 synergistically enhances OXA's anti-cancer effects in CRC cells

Previous studies have shown that PF-309 has a synergistic effect with 5-fluorouracil.^8^ Therefore, we aimed to investigate whether PF-309 also exhibited a synergistic effect with OXA, another commonly used chemotherapeutic drug, for CRC. First, we used the online SynergyFinder software to analyze the combination in HCT116 cells, which yielded an HSA synergy score of 7.069, indicating a high level of synergy (Fig. 6A). Based on this analysis, we determined the optimal concentration for OXA to be 3125 nM and for PF-309 to be 1.53 nM (Fig. 6B). Subsequently, we conducted a CCK-8 proliferation assay to measure cell viability, which showed that the combination of drugs further reduced cell proliferation compared with each drug alone (Fig. 6C). Additionally, a colony formation assay demonstrated that the combination treatment significantly decreased the number of colonies formed (Fig. 6D). To confirm that the observed anti-migration and anti-invasion effects were not due to reduced cell proliferation, we used serum-free medium in the upper chamber of the transwell assay, eliminating the influence of cell proliferation on migration and invasion. These results demonstrated that the combination treatment effectively inhibited cell migration and invasion independently of its effects on proliferation (Fig. 6E). Similar experiments were conducted using DLD1 cells. The SynergyFinder analysis for DLD1 cells yielded an HSA synergy score of 16.295, with the optimal combination concentrations determined to be 25000 nM for OXA and 781.25 nM for PF-309 (Fig. S4A, B). The combination treatment of OXA and PF-309 significantly reduced cell proliferation, colony formation, and cell migration and invasion, respectively (Fig. S4C–E). To further validate the synergy between PF-309 and OXA, we tested the combination therapy on two CRC patient organoids, P01 and P02. The combined treatment significantly reduced organoid size and viability compared with each drug alone (Fig. 6F), confirming the synergistic effect seen in cell lines and showing its effectiveness in 3D culture systems.

**Figure 6:**
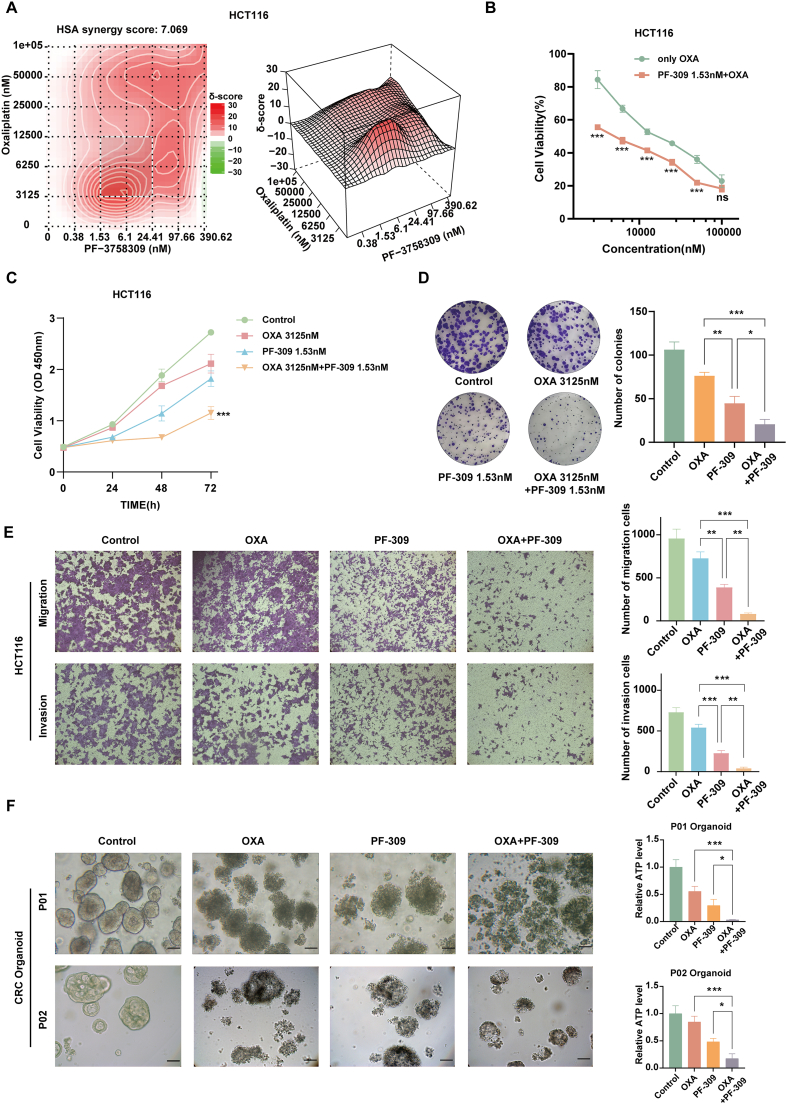
Synergistic effects of PF3758309 (PF-309) and oxaliplatin (OXA) in HCT116 cells. **(A)** Synergy analysis of PF-309 and OXA combination using SynergyFinder software in HCT116 cells. The HSA synergy score of 7.069 indicates a moderate level of synergy. **(B)** Determination of optimal concentrations for OXA (3125 nM) and PF-309 (1.53 nM) based on SynergyFinder analysis. **(C)** CCK-8 proliferation assay results show the cell viability of HCT116 cells treated with optimal concentrations of OXA, PF-309, and their combination over three days, measured by absorbance at 450 nm. The combination treatment significantly reduced cell proliferation. **(D)** Colony formation assay demonstrated that the combination treatment significantly decreased the number of colonies formed in HCT116 cells. **(E)** Transwell assay results indicate that the combination of PF-309 and OXA could significantly inhibit the migration and invasion of HCT116 cells. **(F)** Effect of PF-309 and OXA combination on CRC organoids. Treatment with PF-309, OXA, or their combination was applied to two CRC organoid models (P01 and P02). The combination treatment significantly reduced organoid size and viability, as indicated by ATP levels, compared with each drug alone. The scale bar represents 100 μm. The data were presented as mean ± standard deviation, with statistical significance indicated (ns, no significance; ∗*p* < 0.05, ∗∗*p* < 0.01, and ∗∗∗*p* < 0.001).

In addition, we found that the combination of PF-309 and OXA could further reduce the expression of mTOR, p70 S6K, and CD44 mRNA and protein in HCT116 (Fig. 7A–D) and DLD1 (Fig. S5A–D) cells. To further confirm the mechanism of the combination therapy in CRC, we utilized the SUnSET assay to evaluate protein synthesis activity after treatment with PF-309, OXA, and their combination in HCT116 and DLD1 cells. The combination of PF-309 and OXA significantly reduced puromycin incorporation compared with single treatments, indicating a stronger inhibition of protein synthesis when both agents were used together (Fig. 7E; Fig. S5E). Furthermore, mRNA expression levels of MTOR, CD44, and SAA1 in HCT116 and DLD1 cells were measured using the actinomycin D assay to assess the time-dependent effects of the treatments (Fig. 7F**–**M; Fig. S5F–M). The combination treatment resulted in a faster and more pronounced degradation of MTOR, CD44, and SAA1 mRNA levels compared with either PF-309 or OXA alone, highlighting the accelerated degradation when both agents were used together. In contrast, mRNA levels of U6 and PAK2-6 showed no significant trend across the different treatment groups.

**Figure 7:**
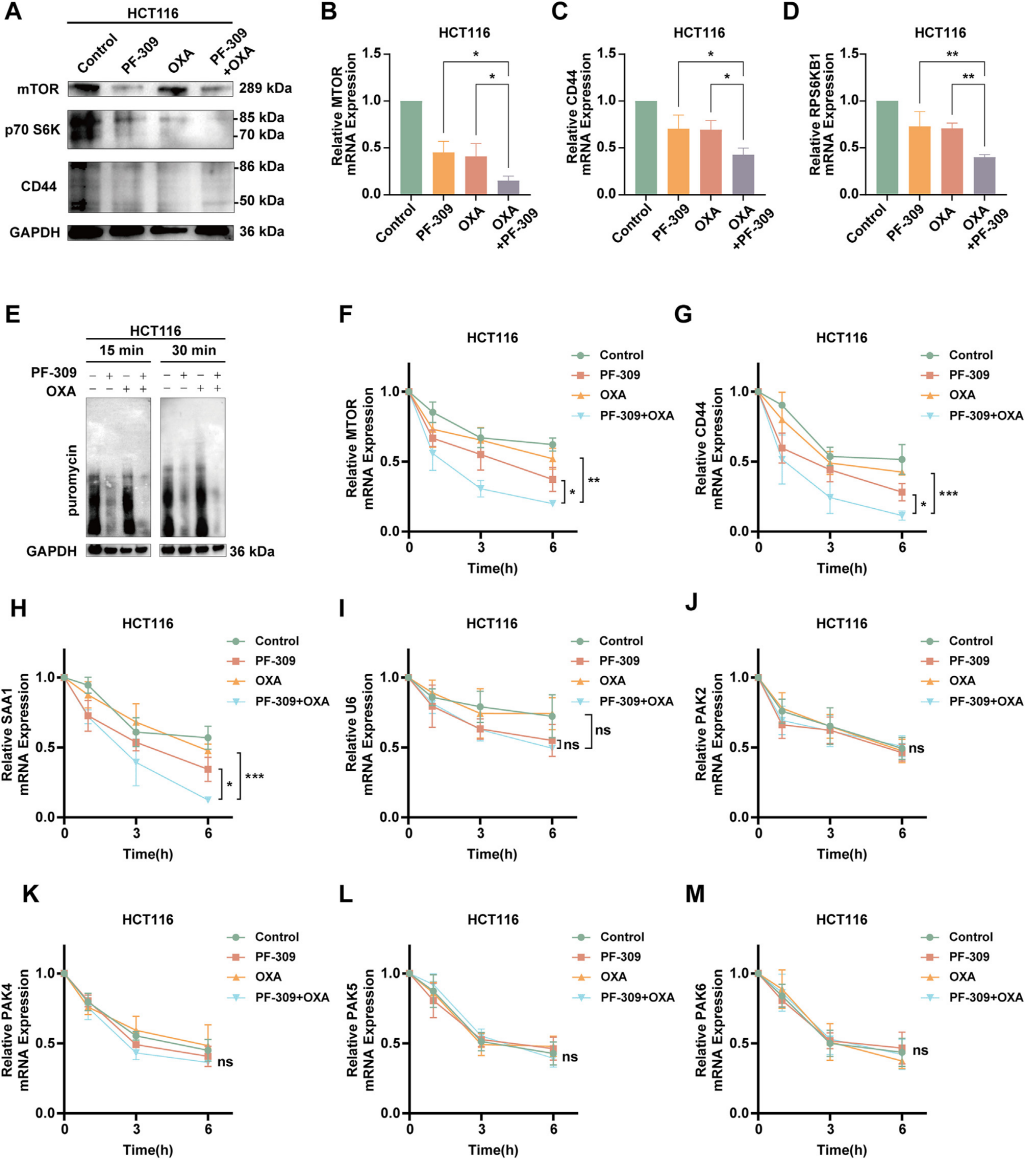
Effects of PF3758309 (PF-309) and oxaliplatin (OXA) combination on signaling pathways and mRNA stability in HCT116 cells. **(A–D)** Western blotting (A) and quantitative real-time PCR (B–D) analysis of mTOR, p70 S6K, and CD44 protein and mRNA levels in HCT116 cells treated with PF-309, OXA, and their combination. The combination treatment led to a significant reduction in the protein and mRNA levels of these key markers compared with individual treatments. **(E)** SUnSET assay showed the effects of PF-309, OXA, and their combination on protein synthesis in HCT116 cells. Cells were treated for either 15 or 30 min, and results indicate that the combination could significantly inhibit protein synthesis. **(F–M)** Relative mRNA expression levels of MTOR (F), CD44 (G), SAA1 (H), U6 (I), PAK2 (J), PAK4 (K), PAK5 (L), and PAK6 (M) in control, PF-309, OXA, and PF-309 plus OXA treated HCT116 cells at 1, 3, and 6 h after actinomycin D treatment. The data were presented as mean ± standard deviation, with statistical significance indicated (ns, no significance; ∗*p* < 0.05, ∗∗*p* < 0.01, and ∗∗∗*p* < 0.001).

To further validate the specificity of PF-309 and confirm that its effects were mediated through PAK1 inhibition, we used another allosteric PAK1 inhibitor, IPA-3, in combination with OXA in HCT116 and DLD1 cells (Fig. S6, 7). The HSA synergy scores for the IPA-3 and OXA combination were comparable to those of PF-309, indicating a similar level of synergy (Fig. S6A–F). Western blotting and mRNA expression analyses revealed that the combination with IPA-3 effectively reduced mTOR, CD44, and SAA1 levels, consistent with the effects observed with PF-309 (Fig. S6G–N). Furthermore, in DLD1 cells, we performed an actinomycin D assay to assess mRNA stability. The combination treatment with IPA-3 and OXA in DLD1 cells showed a similar pattern of accelerated degradation of mTOR, CD44, and SAA1 mRNA as observed previously (Fig. S7A–H). However, genes such as U6, PAK2, PAK4, PAK5, and PAK6 remained unaffected, supporting the specificity of the treatment.

### PF-309 demonstrates *in vivo* synergistic efficacy with OXA in CRC

**Figure 8:**
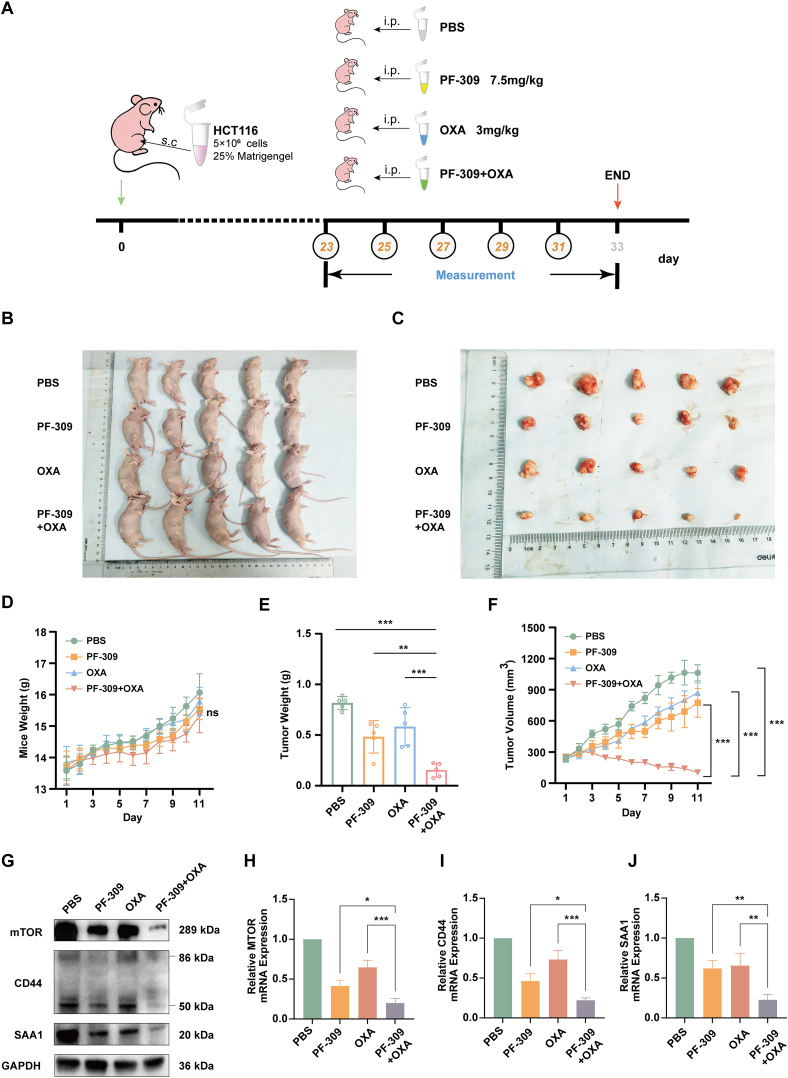
The combination of PF3758309 (PF-309) plus oxaliplatin (OXA) enhances the inhibition of tumor growth *in vivo*. **(A)** The scheme of medication on tumor-bearing mice. **(B)** Images of tumor-bearing mice from each treatment group at the end of the experiment. **(C)** Images of the tumors dissected from the animals. **(D)** Body weight of mice during the treatment period. **(E)** Tumor weight of each group. **(F)** Tumor volume measurements over time in different groups. (G) Western blotting analysis of mTOR, CD44, and SAA1 protein levels in tumor tissues from different treatment groups. **(H–J)** Quantitative real-time PCR analysis of MTOR (H), CD44 (I), and SAA1 (J) mRNA expression levels in tumor tissues from different treatment groups. The data were presented as mean ± standard deviation, with statistical significance indicated (ns, no significance; ∗*p* < 0.05, ∗∗*p* < 0.01, and ∗∗∗*p* < 0.001).

Building on the *in vitro* and organoid findings, we next investigated the synergistic effects of PF-309 and OXA *in vivo*. We established a subcutaneous tumor model by injecting HCT116 cells into nude mice. After tumor implantation, mice were divided into four groups and treated with either phosphate buffer saline, PF-309 (7.5 mg/kg), OXA (3 mg/kg), or a combination of PF-309 and OXA (Fig. 8A). At the end of the treatment period, mice were sacrificed, and tumor images were captured (Fig. 8B, C). The body weight of the mice was monitored throughout the experiment, and no significant weight loss was observed in the combination treatment group compared with the other groups (Fig. 8D). The combination of PF-309 and OXA significantly reduced tumor weight compared with the phosphate buffer saline group and PF-309 and OXA single-treatment groups (Fig. 8E). Furthermore, the tumor volume was significantly smaller in the combination treatment group compared with all other groups (Fig. 8F). Then, we analyzed protein and gene expression in tumor tissues collected from the different treatment groups. Western blotting analysis revealed that mTOR, CD44, and SAA1 protein levels were markedly reduced in the combination treatment group compared with the single-treatment and control groups (Fig. 8G). 10.13039/100014337Furthermore, RT-qPCR analysis showed a significant decrease in the mRNA expression of MTOR, CD44, and SAA1 in the combination group, further supporting the synergistic effect of PF-309 and OXA in inhibiting tumor growth (Fig. 8H**–**J).

Our findings shed light on the potential of targeting PAK1 as a therapeutic strategy in CRC, particularly in combination with OXA, offering new insights for future clinical applications.

## Discussion

Our study has revealed significant insights into the role of PAK1 in CRC and its potential as a therapeutic target. We demonstrate that PAK1 is highly expressed in CRC cell lines compared with normal intestinal epithelial cells, indicating its potential involvement in CRC pathogenesis. Moreover, we showed that PAK1 could promote the activation of mTOR-S6K signaling pathway in CRC cells. Our results have also shed light on the potential of the PAK1 inhibitor PF-309 as a therapeutic agent for CRC. We found that PF-309 exhibited differential sensitivity across various CRC cell lines, suggesting its potential for targeted therapy. Furthermore, our results indicate that PF-309 inhibits key signaling pathways involved in CRC progression, including the mTOR, p70 S6K, EIF4G1, NF-κB, IKB-α, c-Myc, WNT3A, and β-catenin pathways, underscoring its broad spectrum of anti-tumor effects. Additionally, we demonstrated the synergistic effects of PF-309 with the chemotherapeutic drug OXA in CRC cells. We showed that the combination of PF-309 and OXA could significantly reduce cell proliferation, colony formation, migration, and invasion compared with each drug alone, indicating a potential synergistic effect between the two drugs in CRC treatment.

p70 S6K is an important serine/threonine kinase belonging to the S6 kinase family. It plays an important role in cell proliferation, growth, and metabolic regulation. p70 S6K exerts its function mainly by phosphorylating target protein S6, a subunit of ribosomal protein S6, which plays an important role in transcription and translation regulation. p70 S6K activity is regulated by a variety of signaling pathways, including the PI3K/Akt pathway and MAPK pathway.^19^^,^^20^ The abnormal activation of p70 S6K is closely related to the occurrence, development, invasion, and metastasis of tumors. p70 S6K is considered to be an important oncogene whose overactivation can promote tumor-related biological processes such as cell proliferation, growth, and angiogenesis. Therefore, p70 S6K is considered a potential anti-cancer therapeutic target.^21^ We first found that PAK1 activated mTOR-S6K by inhibiting mRNA decay of MTOR. This discovery sheds light on the molecular mechanisms driving CRC progression, providing a potential target for therapeutic intervention. Targeting the PAK1/mTOR/p70 S6K pathway could lead to novel treatments that inhibit cancer growth and metastasis, ultimately improving patient outcomes.

Several studies have shown that PAK1 can interact with and phosphorylate RNA-binding proteins, which are essential for mRNA processing. PAK1-dependent phosphorylation of PCBP1 regulates splicing and translation.^15^ In addition, active PAK1 phosphorylates FXR1, which is then recruited to stress granules.^16^ PAK1 also controls cell shape and polarity by directly phosphorylating the RNA-binding protein Sts5 (orthologue of human DIS3L2) and subsequently dissociating it from P-bodies in fission yeast cells.^17^ These studies indicate that PAK1 might promote CRC progression by regulating mRNA decay. In our study, we further explored the interaction between PAK1 and RNA-binding proteins in CRC cells. Co-immunoprecipitation using DLD1 and HT29 cell lines confirmed that PAK1 interacted with FXR1 (Fig. S8A). To further understand the role of FXR1 in this context, we performed western blotting analysis on DLD1 cells transfected with either negative control (NC) siRNA or FXR1 siRNA (si-FXR1). The results showed that knockdown of FXR1 effectively reduced FXR1 expression but did not significantly affect PAK1 levels (Fig. S8B). However, we observed a decrease in mTOR and SAA1 expression following FXR1 knockdown. This finding supports the hypothesis that PAK1 may influence mRNA stability in CRC by interacting with FXR1. However, the specific mechanisms by which PAK1 and FXR1 cooperate to regulate mRNA stability and their implications for CRC progression remain to be further elucidated in future studies.

CD44 is a highly conserved transmembrane glycoprotein originally found on the surface of lymphocytes. Its role as a cell adhesion molecule is widespread in a variety of cell types and is involved in cell-extracellular matrix interactions.^22^ The CD44 gene is located on human chromosome 11p13 and encodes a protein that is expressed on the surface of cell membranes.^23^ The basic structure of CD44 consists of an N-terminal external domain, a single transmembrane domain, and a C-terminal intracellular domain^24^. CD44 has a variety of isoforms whose functions are involved in cell adhesion, migration, proliferation, and signal transduction. High expression of CD44 in CRC is associated with poor prognosis in patients.^25^ CD44^+^ CSCs have the ability of self-renewal and differentiation, can form new tumors in the body, and have strong resistance to traditional chemotherapy and radiotherapy.^26^ CD44/PAK1/AKT activation may help predict the response to FGFR1 inhibition in squamous cell lung cancer.^27^ In addition, PAK1 has been reported to regulate the alternative splicing of CD44 by phosphorylating PCBP1^15^ or promote the expression of CD44 through the PAK1/ATF2/miR-132 cascade in gastric cancer.^28^ However, we showed that PAK1 could regulate the mRNA stability of CD44 expression in CRC cells.

The role of SAA1 in CRC has attracted much attention. SAA1 is an acute reactive protein whose expression levels are often significantly increased in disease states such as inflammation and tumors.^29^^,^^30^ The expression of SAA1 is closely related to the diagnosis and prognosis of CRC. Multiple studies have shown that high expression of SAA1 is associated with the development of CRC, lymph node metastasis, and poor prognosis. Therefore, detection of SAA1 expression level may be helpful for early diagnosis and prognosis assessment of CRC.^31^ This finding suggests that PAK1 may influence the occurrence and development of CRC by regulating the expression of SAA1. Furthermore, we found that PAK1 regulated the mRNA stability of SAA1 expression in CRC cells. Thus, our results indicate that PAK1 might specifically regulate the mRNA stability of the secreted protein.

PAK inhibitor PF-309 suppressed xenograft CRC cell growth in SCID mice by inhibiting PAK1 activity.^8^ Moreover, PAK1 inhibition synergizes with 5-fluorouracil therapy, indicating the possibility of combining PAK1 inhibitors with conventional 5-fluorouracil-based chemotherapy for CRC.^8^ Our study revealed that PF-309 could synergize with OXA, another key drug in CRC treatment, introducing a novel approach to CRC therapy.

In summary, our study elucidated the novel oncogenic mechanism of PAK1 in CRC progression. Loss of PAK1 promoted mRNA decay and inhibited the mRNA stability of multiple oncogenic factors, including CD44, SAA1, MTOR, RPS6KB1, and EIF4G1. Importantly, our study reveals that the PAK1 inhibitor PF-309 exhibits a synergistic effect with OXA in CRC. Collectively, our results highlight the potential of targeting PAK1 as a therapeutic strategy in CRC, particularly in combination with OXA.

## CRediT authorship contribution statement

**Rongtian Pan:** Writing – review & editing, Writing – original draft, Visualization, Validation, Formal analysis, Data curation. **Renrui Zou:** Writing – review & editing, Writing – original draft, Formal analysis, Data curation. **Ying Sui:** Writing – review & editing, Writing – original draft, Data curation. **Fei Wu:** Writing – review & editing, Writing – original draft, Data curation. **Dongfeng Wang:** Project administration, Methodology, Investigation. **Yuan Zhang:** Writing – review & editing, Writing – original draft, Visualization, Validation, Supervision, Project administration, Methodology, Formal analysis, Data curation, Conceptualization. **Shaorong Yu:** Writing – review & editing, Writing – original draft, Visualization, Validation, Supervision, Software, Resources, Project administration, Methodology, Investigation, Funding acquisition, Formal analysis, Data curation, Conceptualization.

## Ethics declaration

Animal welfare and ethical reviews for experiments were conducted by the Institutional Animal Care and Use Committee of Nanjing Medical University (Approval No. IACUC-2312046). Ethical consent was approved by the Committees for Ethical Review of Research involving Human Subjects at Jiangsu Cancer Hospital.

## Data availability

The raw data and materials supporting the conclusions of this article are available from the corresponding author upon reasonable request.

## Funding

This study was supported by grants from the National Natural Science Foundation of China (Nos. 81902489 and 82172872), the Yishan Research Project of Jiangsu Cancer Hospital (No. YSZD/PY202408), the Key Research and Development Program of Jiangsu Province (BE2021745), and the Natural Science Foundation of Jiangsu Province (BK20191079).

## Conflict of interests

The authors declared no conflict of interests.

## Figures and Tables

**figs1:**
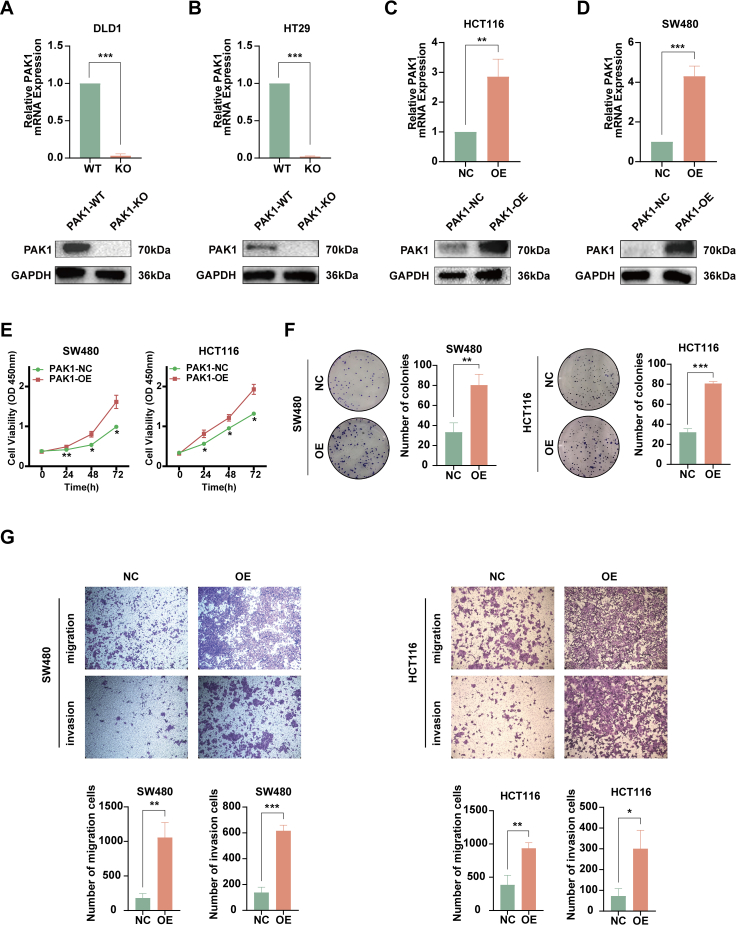


**figs2:**
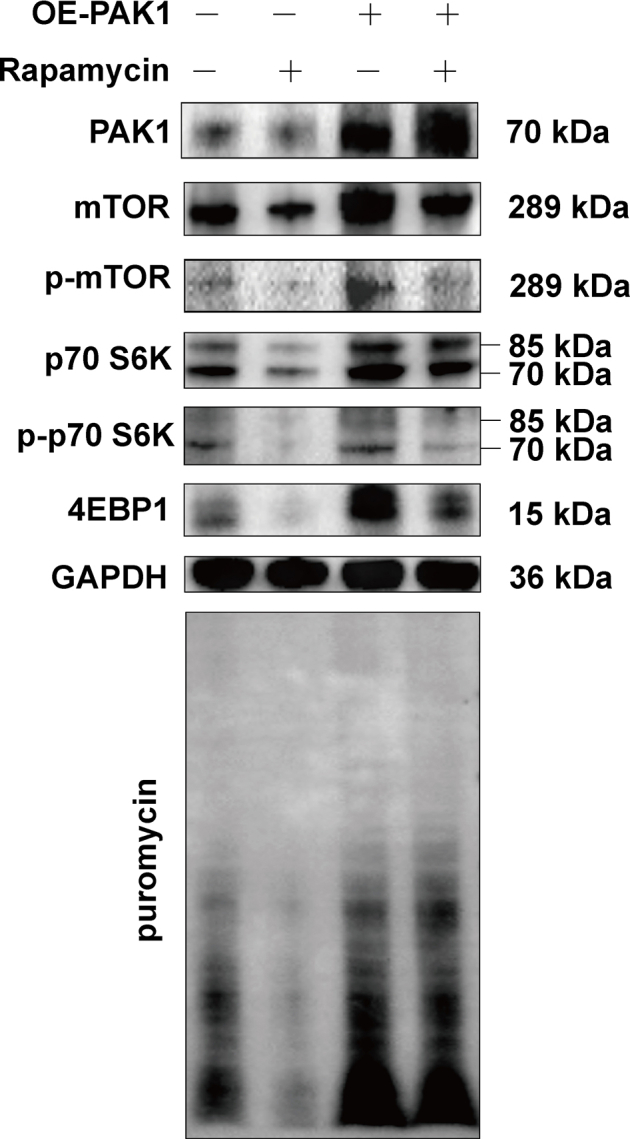


**figs3:**
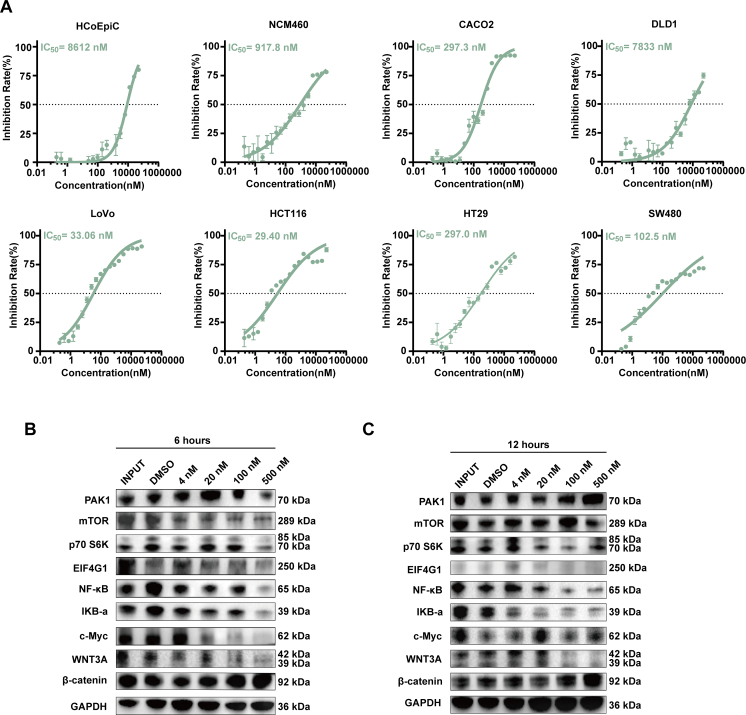


**figs4:**
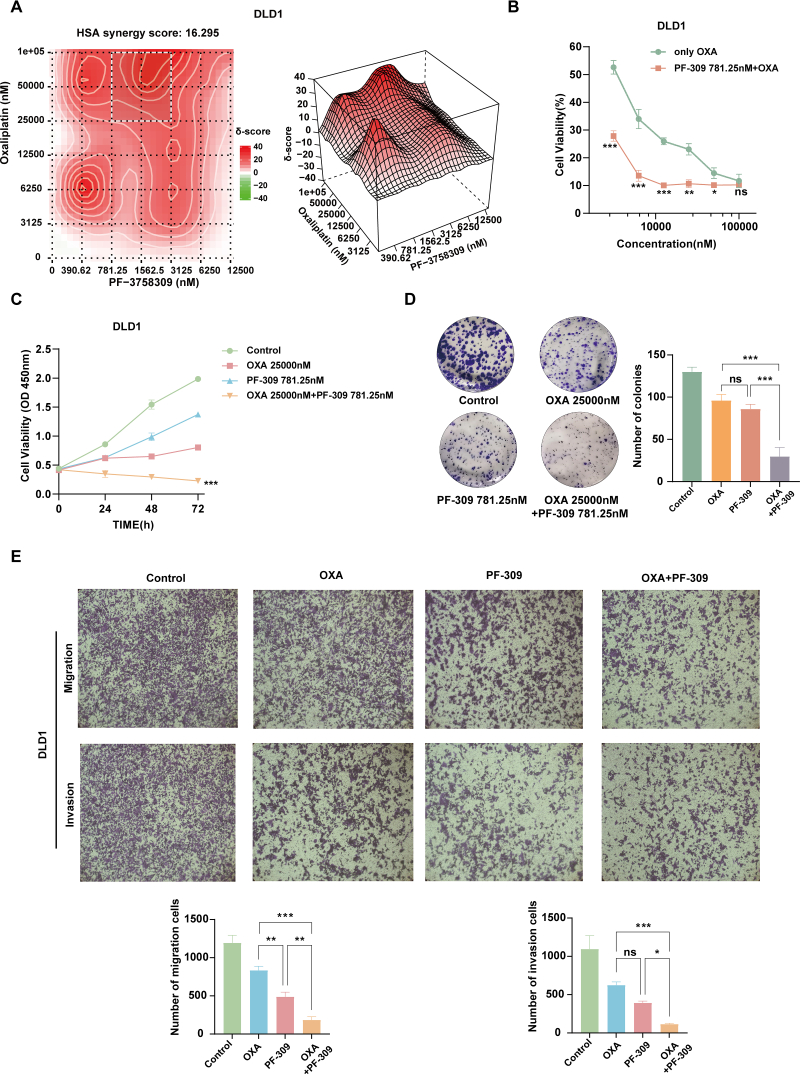


**figs5:**
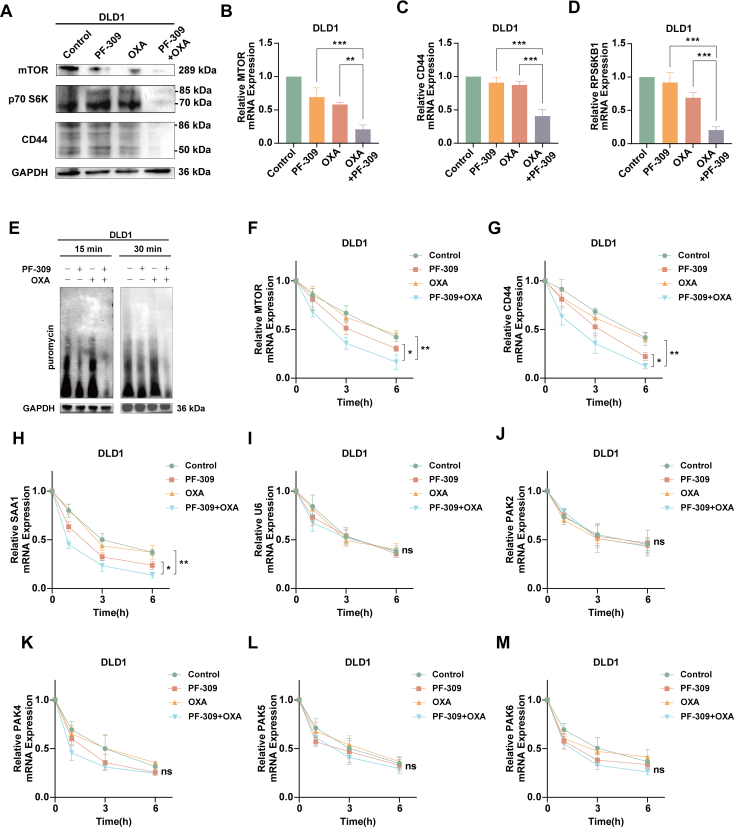


**figs6:**
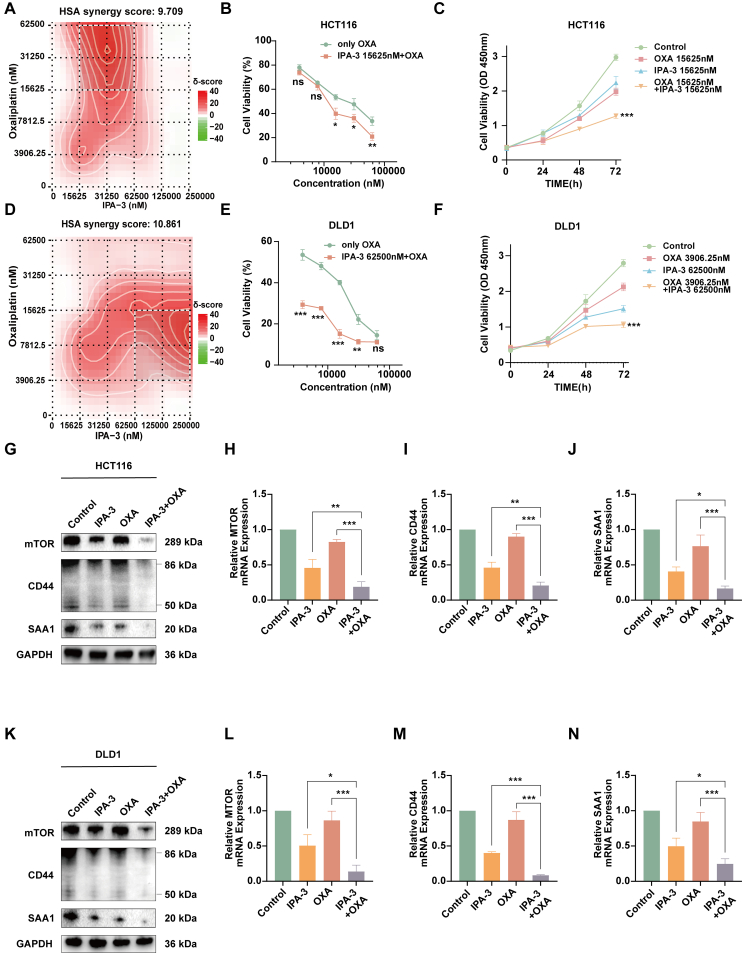


**figs7:**
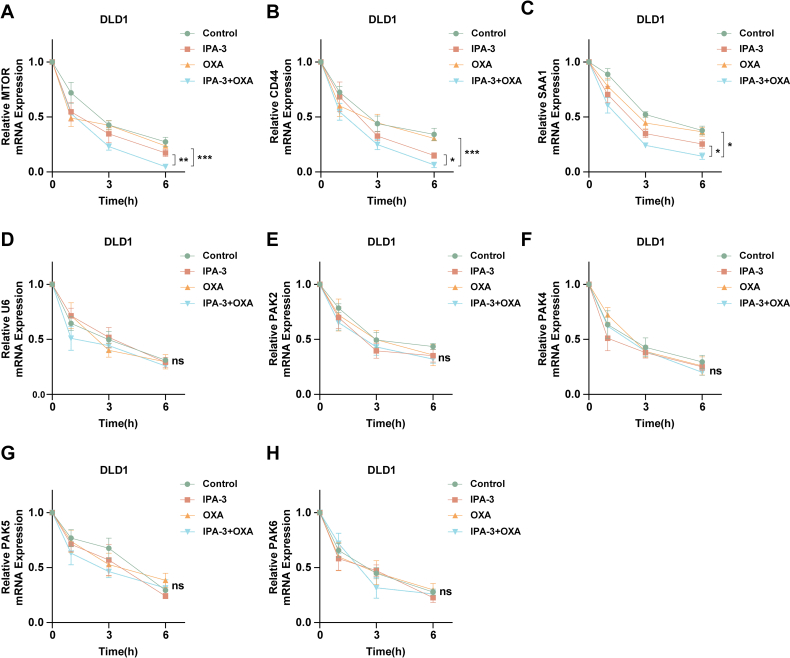


**figs8:**