# Supplementary material for: Targeting PAK1 suppresses tumor progression by promoting mRNA decay of oncogenic factors and enhancing chemotherapeutic efficacy in colorectal cancer
Source: Genes Dis. 2025 May 11;13(2):101683. doi: 10.1016/j.gendis.2025.101683 (PMC12741398; doi:10.1016/j.gendis.2025.101683)

**Supplementary Data**

- **Supplementary tables**

**Table S1** siRNA sequences used in the study.

**Table S2** list of antibodies used in the study.

**Table S3** primer sequences used for quantitative RT-PCR.

- **Supplementary figures**

**Figure S1** PAK1 deficiency inhibits CRC progression. **(A-B)** Validation of PAK1 KO efficiency in DLD1 (A) and HT29 (B) cells. Relative PAK1 mRNA and protein expression was measured by RT-qPCR and WB. **(C-D)** Validation of PAK1 OE efficiency in HCT116 (C) and SW480 (D) cells. Relative PAK1 mRNA and protein expression was measured by RT-qPCR and WB. **(E)** Cell viability assay showing the proliferation of SW480 (left) and HCT116 (right) cells with PAK1 OE compared to NC over time (0, 24, 48, 72 hours). **(F)** Colony formation assay of SW480 (left) and HCT116 (right) cells with PAK1 OE compared to NC. Representative images of colonies and quantification of the number of colonies are shown. **(G)** Transwell migration and invasion assays of SW480 (left) and HCT116 (right) cells with PAK1 OE compared to NC. Representative images of migrated and invaded cells (upper panels) and quantification of the number of migrated and invaded cells (lower panels) are shown. Data are presented as mean ± SD, with statistical significance indicated (* for *P* < 0.05, ** for *P* < 0.01, and *** for *P* < 0.001).

**Figure S2** Effects of PAK1 overexpression and rapamycin on mTOR pathways. WB analysis of HCT116 cells showing mTOR, p-mTOR, p70 S6K, p-p70 S6K, and 4EBP1 levels under PAK1 overexpression (OE-PAK1) and rapamycin treatment. The puromycin signal in OE-PAK1 cells remained consistent even with mTOR inhibition.

**Figure S3** Efficacy of PF3758309 (PF-309) in CRC cell lines and its impact on signaling pathways. **(A)** Determination of IC_50_ of PF-309 in two normal intestinal epithelial cell lines (HCoEpiC and NCM460) and six CRC cell lines (CACO2, DLD1, HT29, HCT116, LoVo, and SW480). The IC_50_ values varied across the cell lines, indicating differential sensitivity to the inhibitor. **(B-C)** WB analysis of HCT116 cells treated with PF-309 to explore its impact on various signaling pathways. Treatment with PF-309 for 6 (Fig. S3B) and 12 (Fig. S3C) hours resulted in the inhibition of several key pathways, including the mTOR, p70 S6K, EIF4G1, NF-κB, IKB-α, c-Myc, WNT3A, and β-catenin pathways.

**Figure S4** Synergistic effects of PF3758309 (PF-309) and Oxaliplatin (OXA) in DLD1 cells. **(A) Synergy analysis of PF-309 and OXA combination using SynergyFinder software in DLD1 cells. The HSA synergy score of 16.295 indicates a high level of synergy. (B) Determination of optimal concentrations for OXA (25000 nM) and PF-309 (781.25 nM) based on SynergyFinder analysis. (C) CCK-8 proliferation assay results showing cell viability of DLD1 cells treated with optimal concentrations of OXA, PF-309 and their combination over three days, measured by absorbance at 450 nm. The combination treatment significantly reduced cell proliferation compared to each drug alone. (D) Colony formation assay demonstrating that the combination treatment significantly decreased the number of colonies formed in DLD1 cells. (E) Transwell assay results indicating that the combination of PF-309 and OXA significantly inhibited the migration and invasion of DLD1 cells.** Data are presented as mean ± SD, with statistical significance indicated (ns for no significance, * for *P* < 0.05, ** for *P* < 0.01 and *** for *P* < 0.001).

**Figure S5 Effects of PF3758309 (PF-309) and Oxaliplatin (OXA) combination on signaling pathways and mRNA stability in DLD1 cells. (A-D) WB (A) and RT-qPCR (B-D) analysis of mTOR, p70 S6K, and CD44 protein levels in DLD1 cells treated with PF-309, OXA and their combination. The combination treatment led to a significant reduction in the protein levels of these key markers compared to individual treatments. (E) SUnSET assay showing the effects of PF-309, OXA and their combination on protein synthesis in DLD1 cells. Cells were treated for either 15 or 30 minutes, and results indicate that the combination significantly inhibited protein synthesis. (F-H)** Relative mRNA expression levels of mTOR (F), CD44 (G), SAA1 (H), U6 (I), PAK2 (J), PAK4 (K), PAK5 (L) and PAK6 (M) in control, PF-309, **OXA** and PF-309 + **OXA** treated **DLD1** cells at 1, 3, and 6 hours after Actinomycin D treatment. Data are presented as mean ± SD, with statistical significance indicated (ns for no significance, * for *P* < 0.05, ** for *P* < 0.01 and *** for *P* < 0.001).

**Figure S6** Synergistic effects of IPA-3 and Oxaliplatin (OXA) in HCT116 and DLD1 cells. **(A, D): Synergy analysis of the combination of IPA-3 and OXA using SynergyFinder software in HCT116 (A) and DLD1 (D) cells. The HSA synergy scores I ndicate a strong synergistic interaction in both cell lines. (B, E) Dose-response curves showing the cell viability of HCT116 (B) and DLD1 (E) cells treated with OXA alone or in combination with IPA-3 at the indicated concentrations. The combination treatment significantly reduced cell viability compared to OXA alone. (C, F) CCK-8 proliferation assay results showing the effects of OXA, IPA-3 and their combination on HCT116 (C) and DLD1 (F) cell proliferation over 72 hours. The combination treatment demonstrated a more substantial inhibition of cell proliferation. (G, K) WB analysis of mTOR, CD44, and SAA1 protein levels in HCT116 (G) and DLD1 (K) cells under different treatments. The combination of IPA-3 and OXA significantly reduced these protein levels compared to single treatments. (H-J, L-N) Quantitative analysis of mRNA levels for mTOR (H, L), CD44 (I, M), and SAA1 (J, N) in HCT116 and DLD1 cells, showing that the combination treatment resulted in a greater reduction in mRNA levels compared to each treatment alone.** Data are presented as mean ± SD, with statistical significance indicated (ns for no significance, * for *P* < 0.05, ** for *P* < 0.01 and *** for *P* < 0.001).

**Figure S7** Actinomycin D assay results in DLD1 cells treated with IPA-3 and Oxaliplatin (OXA). **(A-C)** mRNA degradation curves for mTOR (A), CD44 (B), and SAA1 (C) in DLD1 cells treated with IPA-3, OXA or their combination over a 6-hour period. The combination treatment accelerated the degradation of these mRNAs compared to single treatments. **(D-H)** mRNA expression levels of U6 (D), PAK2 (E), PAK4 (F), PAK5 (G) and PAK6 (H) in DLD1 cells across different treatments. No significant changes were observed, indicating the specificity of the combination effect on mTOR, CD44, and SAA1. Data are presented as mean ± SD, with statistical significance indicated (ns for no significance, * for *P* < 0.05, ** for *P* < 0.01 and *** for *P* < 0.001).

**Figure S8** Analysis of PAK1 and FXR1 interaction in DLD1 and HT29 cells. **(A)** Co-IP analysis demonstrating the interaction between PAK1 and FXR1 in DLD1 (top panel) and HT29 (bottom panel) cells. **(B)** WB analysis of FXR1, PAK1, mTOR, and SAA1 protein expression levels in DLD1 cells transfected with either negative control (NC) siRNA or FXR1 siRNA (si-FXR1). Compared to the NC group, si-FXR1 effectively reduced FXR1 expression but did not significantly affect PAK1 levels. However, knockdown of FXR1 led to a decrease in mTOR and SAA1 expression.

**Table S1 siRNA sequences used in the study**

| **Name** | **Sense Sequence (5’-3’)** | **Antisense Sequence(5’-3’)** |
| --- | --- | --- |
| si-FXR1-1 | A.A.A.U.A.U.A.G.G.U.A.G.U.G.C.A.G.A.A.U.U | U.U.C.U.G.C.A.C.U.A.C.C.U.A.U.A.U.U.U.U.U |
| si-FXR1-2 | G.A.G.A.U.U.A.U.A.U.G.G.U.G.G.C.A.A.A.U.U | U.U.U.G.C.C.A.C.C.A.U.A.U.A.A.U.C.U.C.U.U |
| si-FXR1-3 | U.G.G.U.A.A.C.A.C.U.U.C.A.G.A.A.A.U.A.U.U | U.A.U.U.U.C.U.G.A.A.G.U.G.U.U.A.C.C.A.U.U |

**Table S2 list of antibodies used in the study**

| **Name** | **Catalog Number** | **Manufacturer** |
| --- | --- | --- |
| PAK1 | 21401-1-AP | ProteinTech, China |
| GAPDH | 60004-1-Ig | ProteinTech, China |
| mTOR | 66888-1-Ig | ProteinTech, China |
| p-mTOR | 67778-1-Ig | ProteinTech, China |
| p70 S6K | 66638-1-Ig | ProteinTech, China |
| p-p70 S6K | 28735-1-AP | ProteinTech, China |
| EIF4G1 | 15704-1-AP | ProteinTech, China |
| 4EBP1 | 60246-1-Ig | ProteinTech, China |
| NF-κB | 8242 | Cell Signaling Technology, USA |
| IKB-α | 4812 | Cell Signaling Technology, USA |
| c-Myc | 10828-1-AP | ProteinTech, China |
| CD44 | 15675-1-AP | ProteinTech, China |
| SAA1 | A1655 | Abclone, China |
| β-catenin | 51067-2-AP | ProteinTech, China |
| WNT3A | 26744-1-AP | ProteinTech, China |
| FXR1 | 13194-1-AP | ProteinTech, China |
| Anti-rabbit IgG | 7074 | Cell Signaling Technology, USA |
| Anti-mouse IgG | 7076 | Cell Signaling Technology, USA |

**Table S3 primer sequences used for quantitative RT-PCR**

| **Primers** | **Primer sequences (5’-3’)** |
| --- | --- |
| PAK1 Forward | GAACCACTTCCTGTCACTCCAACTC |
| PAK1 Reverse | TCTCCCACGAGGTAACTGTCCAAG |
| PAK2 Forward | CCGCAGTAGTGACAGAGGAGGAG |
| PAK2 Reverse | GTCCCAGTGCAACGTCAGTAGC |
| PAK4 Forward | CAGCACGAGAATGTGGTGGAGATG |
| PAK4 Reverse | CGTAGGGAAGGCGGGAGATGAG |
| PAK5 Forward | TCTCGGTGACTCGCTCCAACTC |
| PAK5 Reverse | GCTGCGTGGCTGCCTCTATAATAC |
| PAK6 Forward | AGCCCTCTGACCACTTCGGATAC |
| PAK6 Reverse | CTGCTTCCTGAGGTCCATCATCTTG |
| MTOR Forward | ACCTGATGCTGGACCGTCTGAG |
| MTOR Reverse | ATCGCTTGTTGCCTTTGGTATTTGTG |
| RPS6KB1 Forward | GGGAAGAACTTCTGGCTCGAAAGG |
| RPS6KB1 Reverse | TCATTGTCACATCCATCTGCTCTATGC |
| EIF4G1 Forward | CTCACGACTCACCAAGATCACCAAG |
| EIF4G1 Reverse | CTCTACTCCGCTCCTCCACTTCC |
| CD44 Forward | TGCTACTTCAGACAACCACAAGGATG |
| CD44 Reverse | ATGAGGGGAGGGTGTGCTTCTG |
| SAA1 Forward | CCGATGCCAGAGAGAATATCCAGAG |
| SAA1 Reverse | TTTGCCACTCCTGCCCCATTC |
| GAPDH Forward | CAAGGCTGTGGGCAAGGTCATC |
| GAPDH Reverse | GTGTCGCTGTTGAAGTCAGAGGAG |
| U6 Forward | GCTTCGGCAGCACATATACTAAAAT |
| U6 Reverse | CGCTTCACGAATTTGCGTGTCAT |

**

**

**
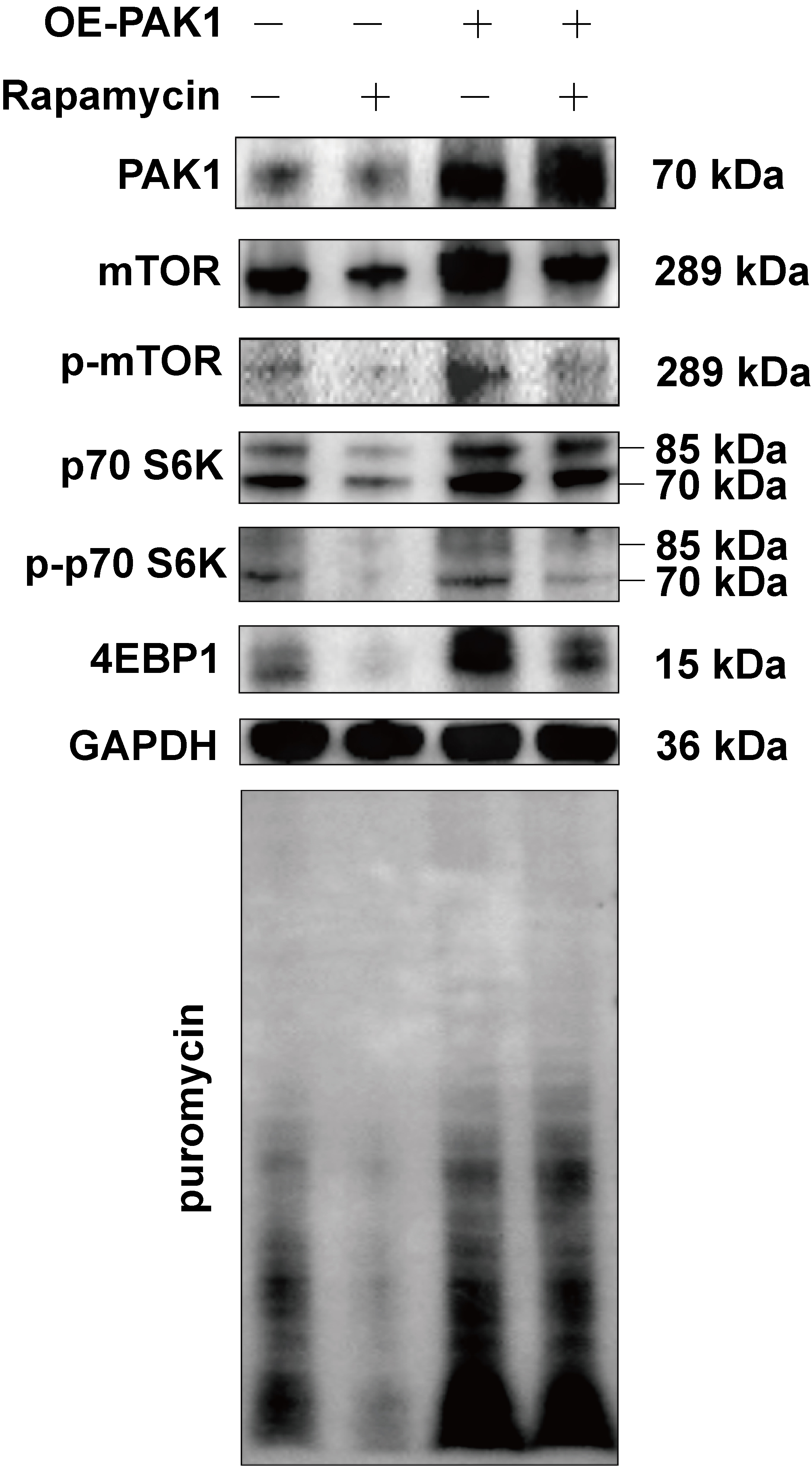
**














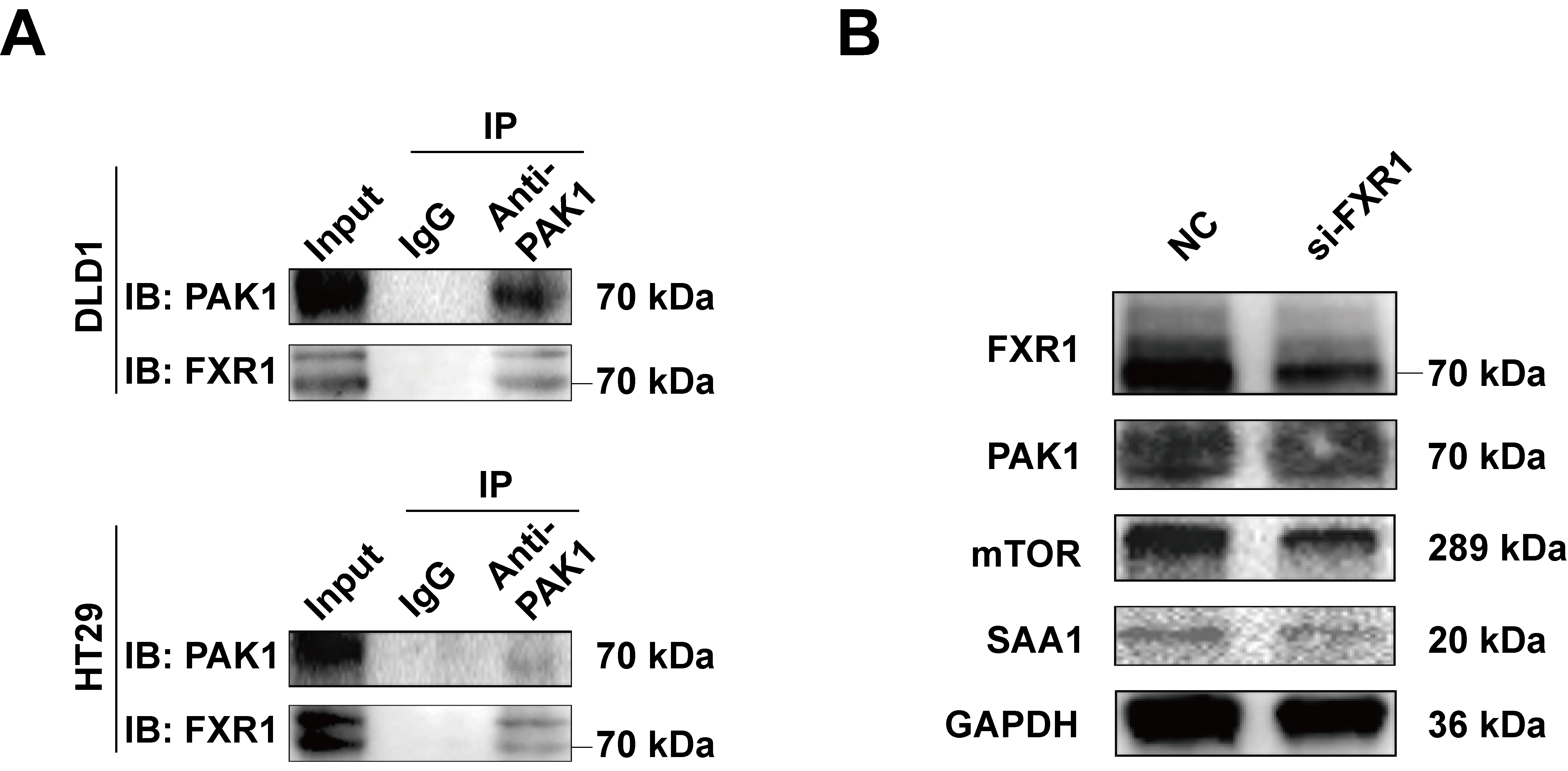

Supplement: Multimedia component 1 [file mmc1.docx]
